# Gill and Liver Transcript Expression Changes Associated With Gill Damage in Atlantic Salmon (*Salmo salar*)

**DOI:** 10.3389/fimmu.2022.806484

**Published:** 2022-03-28

**Authors:** Mohamed Emam, Albert Caballero-Solares, Xi Xue, Navaneethaiyer Umasuthan, Barry Milligan, Richard G. Taylor, Rachel Balder, Matthew L. Rise

**Affiliations:** ^1^ Department of Ocean Sciences, Memorial University of Newfoundland, St. John’s, NL, Canada; ^2^ Cermaq Canada, Campbell River, BC, Canada; ^3^ Cargill Animal Nutrition and Health, Elk River, MN, United States

**Keywords:** moderate gill damage, environmental stressors, transcriptomic response, wound healing, immune response

## Abstract

Gill damage represents a significant challenge in the teleost fish aquaculture industry globally, due to the gill’s involvement in several vital functions and direct contact with the surrounding environment. To examine the local and systemic effects accompanying gill damage (which is likely to negatively affect gill function) of Atlantic salmon, we performed a field sampling to collect gill and liver tissue after several environmental insults (e.g., harmful algal blooms). Before sampling, gills were visually inspected and gill damage was scored; gill scores were assigned from pristine [gill score 0 (GS0)] to severely damaged gills (GS3). Using a 44K salmonid microarray platform, we aimed to compare the transcriptomes of pristine and moderately damaged (i.e., GS2) gill tissue. Rank Products analysis (5% percentage of false-positives) identified 254 and 34 upregulated and downregulated probes, respectively, in GS2 compared with GS0. Differentially expressed probes represented genes associated with functions including gill remodeling, wound healing, and stress and immune responses. We performed gill and liver qPCR for all four gill damage scores using microarray-identified and other damage-associated biomarker genes. Transcripts related to wound healing (e.g., *neb* and *klhl41b*) were significantly upregulated in GS2 compared with GS0 in the gills. Also, transcripts associated with immune and stress-relevant pathways were dysregulated (e.g., downregulation of *snaclec 1-like* and upregulation of *igkv3*) in GS2 compared with GS0 gills. The livers of salmon with moderate gill damage (i.e., GS2) showed significant upregulation of transcripts related to wound healing (i.e., *chtop*), apoptosis (e.g., *bnip3l*), blood coagulation (e.g., *f2* and *serpind1b*), transcription regulation (i.e., *pparg*), and stress-responses (e.g., *cyp3a27*) compared with livers of GS0 fish. We performed principal component analysis (PCA) using transcript levels for gill and liver separately. The gill PCA showed that PC1 significantly separated GS2 from all other gill scores. The genes contributing most to this separation were *pgam2*, *des*, *neb*, *tnnt2*, and *myom1.* The liver PCA showed that PC1 significantly separated GS2 from GS0; levels of *hsp70*, *cyp3a27*, *pparg*, *chtop*, and *serpind1b* were the highest contributors to this separation. Also, hepatic acute phase biomarkers (e.g., *serpind1b* and *f2*) were positively correlated to each other and to gill damage. Gill damage-responsive biomarker genes and associated qPCR assays arising from this study will be valuable in future research aimed at developing therapeutic diets to improve farmed salmon welfare.

## 1 Introduction

**Figure 1:**
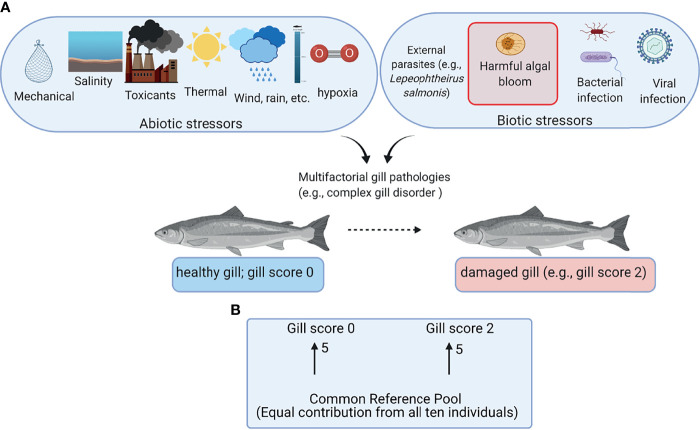
Overview of the field trial and the microarray experimental design. **(A)** Abiotic and biotic stressors potentially contributing to gill damage of farmed Atlantic salmon. **(B)** Common reference design microarray experiment. Arrows represent microarrays with the numbers of biological replicates shown next to the arrows. The base of the arrow shows the Cy3-labeled sample (i.e., common reference pool), and the arrowhead shows the Cy5-labeled sample (i.e., experimental sample). This figure was constructed using BioRender (https://biorender.com/).

Aquaculture is considered as one of the proposed food production sectors capable of filling the current and future gaps between production and rising demand for protein owing to the increasing human population (1). Salmon farming is one of the most successful aquaculture industries with high economic importance in several countries (e.g., Norway, Chile, and Canada) (2). Most farmed salmon are raised in open-net pens from smolts to harvestable size. Although the open-net pens provide aquaculture with the advantage of not competing with livestock on land, they may expose the fish to various environmental stressors including abiotic stressors (e.g., changes in temperature and dissolved oxygen) and biotic stressors (e.g., algal blooms and sea lice) (**Figure 1A**) (3–5).

Environmental stress events can damage salmon organs, including the gills, skin, and gut, which are the main mucosal organs in contact with the external environment (6). The teleost fish gill is a multifunctional organ involved in several physiological processes such as oxygen uptake, salt balance, carbon dioxide clearance, and ammonia excretion (7). To perform those functions, the gill has highly vascular, thin, and long structures (lamellae) that directly receive the entire cardiac output (8). The gill epithelium is a semipermeable barrier that controls the flux of both the water and ions (9), normally preventing pathogen entry. Gills have a packed lamellar structure which provides a large surface area (approximately 0.1-0.4 m^2^ per kg of body mass) (8). While the gill structure (e.g., large surface area with high permeability) allows this organ to perform its main functions, it may also allow the entry of some toxins (e.g. resulting from harmful algal blooms) and toxicants (e.g. detergents and industrial chemical effluents) (10). Also, damaged gills due to environmental events may favor the entry and colonization by several fish pathogens (e.g., *Piscichlamydia salmonis* and *Tenacibaculum maritimum*) (11–13), which may cause systemic infections. Counteracting this, teleost fishes have an aggregation of lymphoepithelial cells at the base of primary gill filaments (i.e., gill-associated lymphoid tissue (GIALT)), that is capable of mounting an adaptive immune response (14). However, frequent gill damage might act as a threat regardless of GIALT’s ability to control infection, due to the organ’s high vascular perfusion (8).

Gill disorders are attracting the attention of aquaculture research, especially with the global environmental changes affecting water quality at open-net pen operations (e.g., changes in temperature and dissolved oxygen) (15). Complex gill disease (CGD) is one of the terms describing the clinical signs occurring in the gill, typically from the end of summer to early winter (11). CGD causative agents are proposed as a mixture of environmental insults, pathogenic agents, and farm management practices (11). Any disorder that affects the gill’s health will have deleterious impacts on its function. Although algae exist in the food web throughout the year, they may grow out of control, causing harmful algal blooms (HABs), which might negatively affect the gill’s health (16). For example, *Chrysochromulina polylepis*, *Chaetoceros convolutus*, *Chaetoceros concavicornis* and *Heterosigma akashiwo* can cause damage and clogging of the gills (17, 18). Those effects are more prominent in aquaculture pen-confined fish (with no or limited ability to escape the event) than in open-water areas. In addition, HABs are often accompanied by a lethal reduction of dissolved oxygen. Also, toxins (e.g., brevetoxins), reactive oxygen species (ROS), and hypoxia have been proposed as causes of severe gill damage leading to fish mortality during and after HAB events (19). Gill damage caused by environmental insults such as HABs might be followed by infection [e.g., *T. maritimum* (20)], which could lead to more severe gill damage and disease.

The west coast of Canada [i.e., British Columbia (BC)] is currently an important area for Atlantic salmon aquaculture production. HABs are a leading cause of mortality to cultured salmon in BC (21), with millions of dollars in estimated annual losses (18). It has been reported that *H. akashiwo* was responsible for a bloom in BC that killed 280,000 Atlantic salmon in 2014, and another HAB in 2018 caused the loss of 250,000 Atlantic salmon (22). Also, both *C. convolutus* and *C. concavicornis* were known to cause fish losses in BC (18). Although the occurrence of algal blooms might be regular in BC, in 2015, an algal bloom with unusual characteristics in terms of duration (May to August) and area (from California to Alaska) occurred (23). This draws the attention to the progression of algal bloom events and the concurrent economic losses, which highlights the necessity of studying the associated effects on aquaculture.

Król et al. (8) found that differences in proliferative gill disease (PGD) scores based on macroscopic examination were not associated with gill transcript expression changes; however, they found that gill histopathology (based on microscopic examination) could be used with RNA-seq data to identify differentially expressed genes associated with multifactorial gill disease (8). The integration of RNA-seq Ingenuity Pathway Analysis and the histopathology highlighted mainly two processes, which were immune/inflammatory response and tissue damage and repair (8). It is likely that different environmental stressors induce different gene expression patterns in fish gills (24). Investigating the transcriptomic response of gills damaged by a distinct set of environmental stressors (i.e., stressors at a different geographical location) might help to identify the common molecular mechanisms involved. Also, it could provide a more robust set of biomarkers associated with gill damage. Complementing this with investigating the response of an internal organ that plays key roles in inflammation and acute phase response (APR; i.e., liver) may enhance our understanding of fish systemic response to gill damage and environmental stress. The consortium for Genomic Research on All Salmonids Project (cGRASP)-designed Agilent 44K salmonid oligonucleotide microarray platform (25) was utilized in the current transcriptome profiling. For the present study, we collected samples of gills presenting different degrees of damage (i.e., from intact to severely damaged) from farmed Atlantic salmon of an open-net production site located in BC. We aimed to identify the global gene expression patterns associated with gill damage due to a combination of environmental factors (including HABs) using 44K microarrays. In addition, we used real-time quantitative polymerase chain reaction (qPCR) to investigate the impact of the gill damage on the transcript levels of different APR, stress, and inflammation-relevant biomarker genes in the liver. The current study improves our understanding of the molecular pathways associated with gill damage in fish and will be valuable in future research and development efforts to improve farmed Atlantic salmon welfare.

## 2 Materials and Methods

### 2.1 Case History, Animals, and Sample Collection

Atlantic salmon smolts with an average initial weight of 102 g were stocked into net pens on a commercial aquaculture site on the west coast of Vancouver Island over the first week of March 2017. This population, prior to smolt entry, had screened negative for Infectious Hematopoietic Necrosis Virus (IHNV), Viral Hemorrhagic Septicemia Virus genotype IVa (VHSV IVa), *Renibacterium salmoninarum*, *Yersinia ruckeri*, *Vibrio anguillarum*, *Vibrio ordalii*, *Aeromonas salmonicida*, and *Piscine orthoreovirus* (PRV). This diagnostic screening was part of the license requirement by Fisheries and Oceans Canada (i.e., minimum of 30 fish sampled per smolt group per hatchery).

As part of the company’s water quality management strategy, the personnel at the farming site monitored water temperature, dissolved oxygen saturation, and salinity at 1, 5, 10, and 15 m, twice daily: once in the morning and once in the afternoon (**Supplementary Figure 1**). The daily monitoring of these water quality physical parameters was often accompanied by water sample collection for visual characterization of the phytoplankton community as part of the Harmful Algae Monitoring Program. These water samples were taken at the same depths and times as the water temperature, dissolved oxygen, and salinity measurements. The phytoplankton identification was outsourced to Microthalassia Consultants Inc. Mortalities were recorded daily and classified based on the most probable cause of death. The most relevant categories in terms of the number of dead fish were: “environmental” for all mortalities attributed to environmental stress (e.g., algal blooms, hypoxia events); “ Mouth rot “ for all mortalities with clinical signs suspected to be caused by *T. maritimum* infection [causative agent of ulcerative stomatitis (also called mouth rot disease) in salmonids; note: infection was visually diagnosed]; “non-performers”, for those salmon euthanized due to their poor growth performance; and “old” for all cases where the fish carcass was too deteriorated to be classified. The recorded environmental mortalities were associated with poor gill condition (B. Milligan, personal communication). **Supplementary Figure 2** shows an example of the observed gill condition. The water temperature, dissolved oxygen, and salinity data for the period June 1 - November 26, 2017, phytoplankton identification (at the species level) and concentration (cell/mL) data for the period June 1-October 31, 2017 and the fish mortality records for the period June 1-December 4, 2017, are shown in **Supplementary Figure 1**.

The fish were sampled on the 13^th^ and 14^th^ of November 2017. The gill and liver samples were collected from the net pen-grown Atlantic salmon located in BC. Fish were randomly netted then euthanized using 400 mg L^−1^ tricaine-methane sulfonate bath (TMS; Syndel Laboratories, Nanaimo, BC, Canada) after 24 h of fasting. The sampled fish were weighed, fork length-measured, and their gills were scored following the farm’s protocol (see section *Gill Scoring Method*). Gill and liver samples were collected for gene expression analyses. Five to ten gill filament fragments (~25-30% of the filament length from the tip) were collected from the medial region of the first gill arch on the left side of each salmon. For salmon presenting gill lesions, we sampled gill filaments that had not been severely eroded (i.e., retained ~75% of their original length, based on adjacent intact gill filaments). Samples around 50-100 mg of each tissue were preserved in 1 mL of RNAlater^®^ solution (Ambion, Inc., Austin, TX, USA) and kept at 4°C overnight, then moved to -20°C until the shipping date to the Ocean Sciences Centre, Memorial University.

### 2.2 Gill Scoring Method

Criteria for routine macropathological (i.e. gross) scoring of gill condition in live fish were developed based on previous studies assessing nonspecific gill lesions (e.g., gill scoring in (26, 27)). Gill scores of 0 (i.e., no lesions visible) and 1 (i.e., single necrotic filament or spot; <1% total gill filament surface area may be affected) from references (26, 27) were combined into one score (i.e., GS0) as the reliability between assessors differentiating scores of 0 or 1 (from references (26, 27)) was poor. Gills showing affected filament surface area up to 10% of the total gill filament were scored as GS1, while GS2 was defined as gills having damage affecting ~10% to 25% of the total filament surface area. Gill scores of 4 (i.e., 20-50% of the gill filament surface area damaged) and 5 (i.e., > 50% of the gill filament surface area) from references (26, 27) were combined into one score (i.e., GS3; > 25% of the total fill filament surface area affected) in the current study’s scoring method. Fish with more than 50% of the total gill filament surface area affected were routinely observed but generally in mortalities or very moribund fish and not in healthy fish (B. Milligan, personal communication). The above gill scoring methodology is summarized in **Table 1**.

**Table 1:** Descriptive gill lesions corresponding to each assigned gill score.

Gill score	Percentage (%) of gill surface area affected	Lesion description	Image example
GS0	Less than 1% total gill filament surface area affected	Overall normal-appearing red gill tissue. May have subtle lesions including pale, blunted, or fused gill edges or partial thickening of a few gill filaments if present.	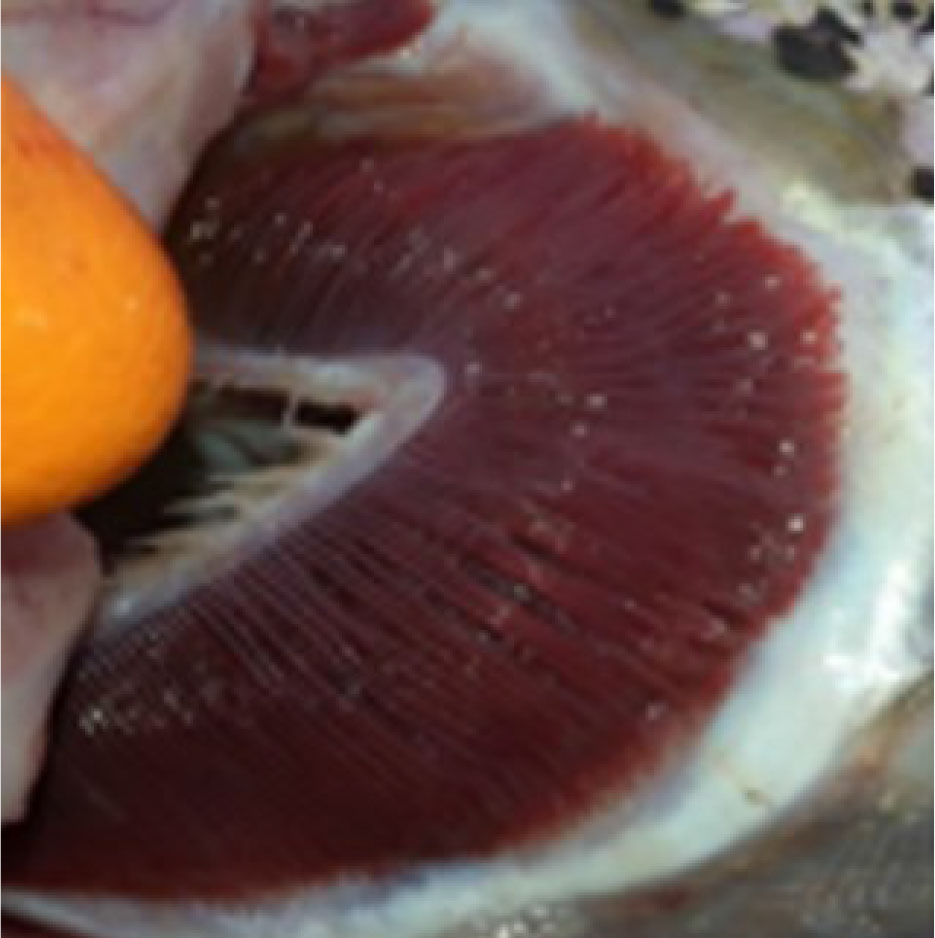
GS1	Between 1% to 10% of total gill filament surface area	Obvious lesions including pale, discolored, or necrotic filaments (short, thickened filaments) affecting between 1 and 10% of total gill filament surface area.	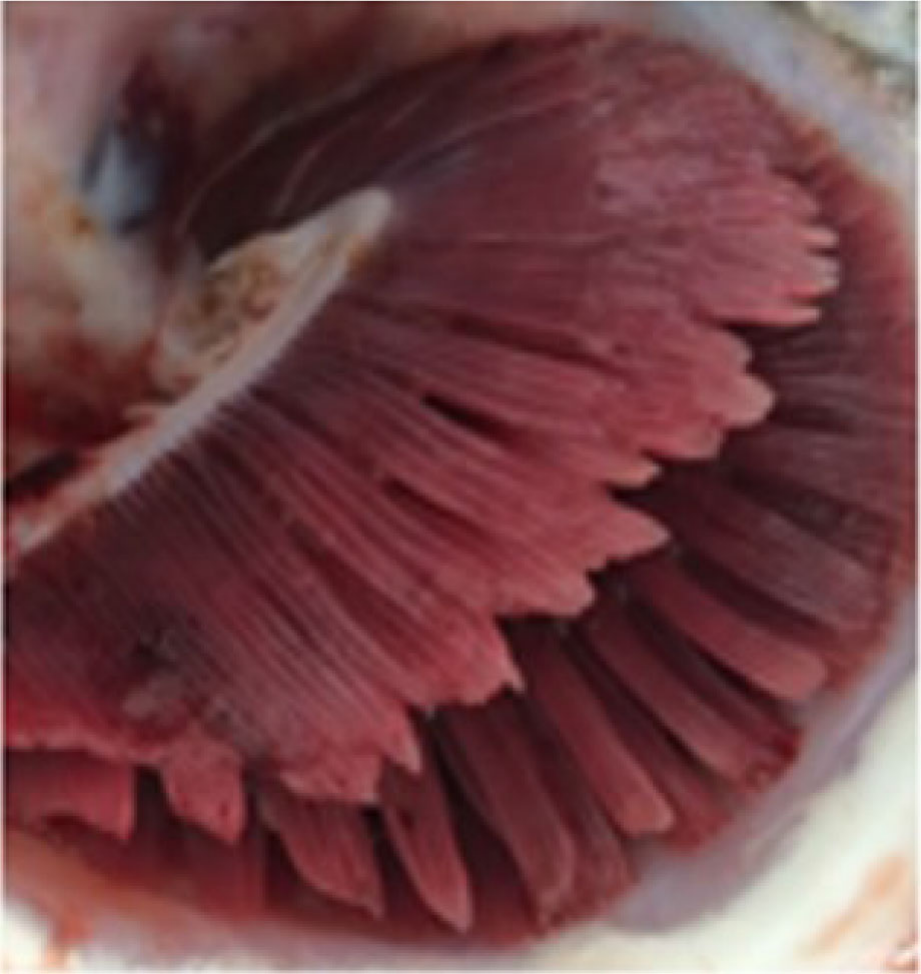
GS2 (Moderately damaged)	Between 10% and 25% of total gill filament surface area	Obvious lesions including pale, discolored, or necrotic filaments (short, thickened filaments) affecting between 10 and 25% of total gill filament surface area.	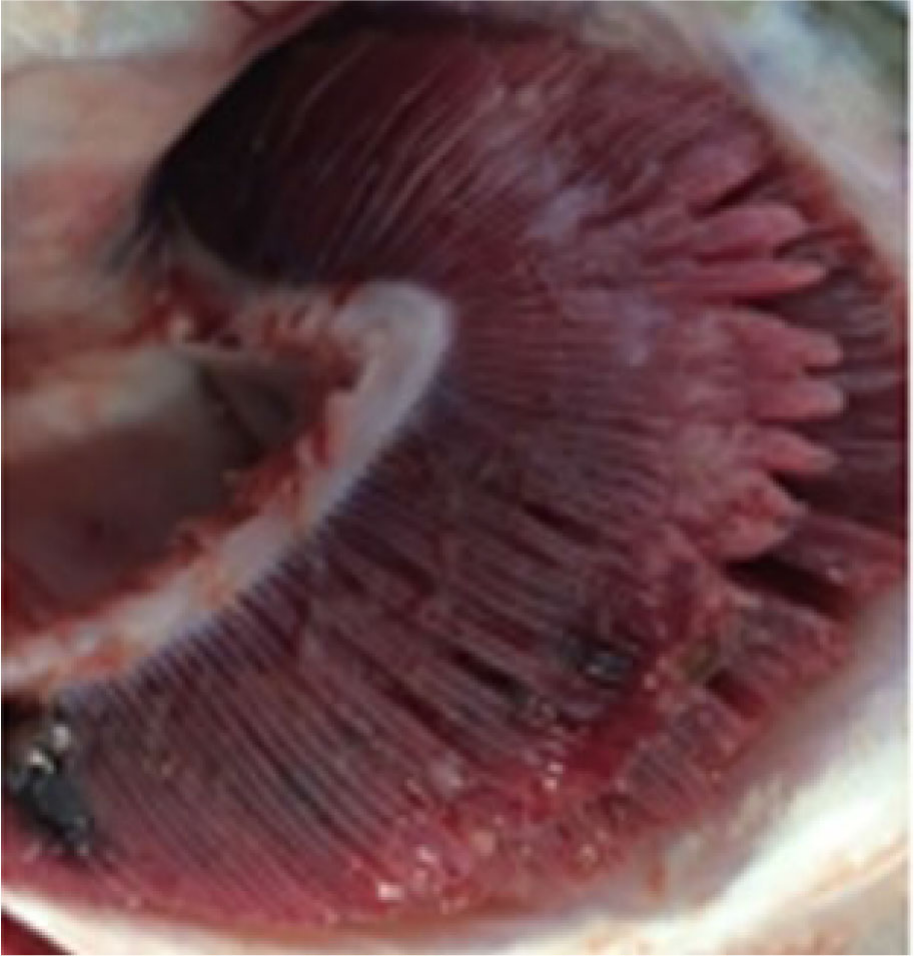
GS3 (Severely damaged)	More than 25% of total gill filament surface area	Obvious lesions including pale, discolored, or necrotic filaments (short, thickened filaments) affecting more than 25% of total gill filament surface area.	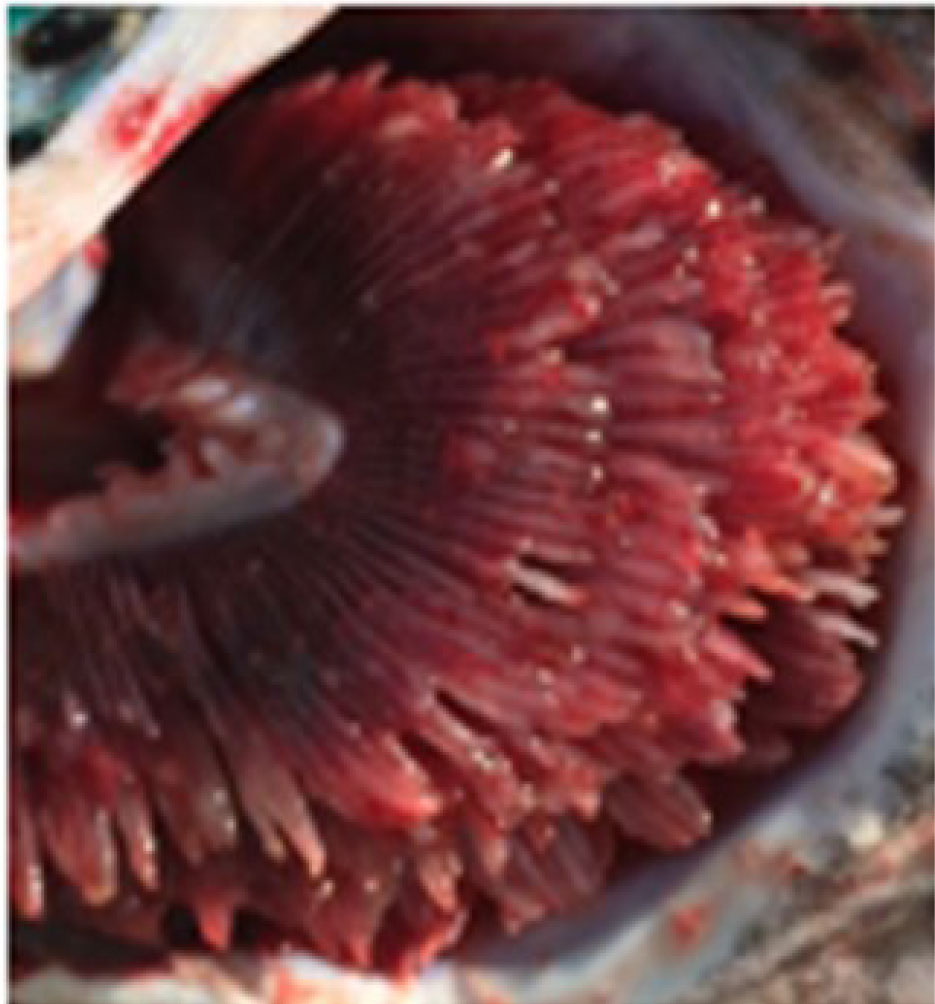

### 2.3 RNA Extraction, DNase Treatment, and Column Purification

Gills were homogenized in 800 µl TRIzol (Invitrogen/Life Technologies, Carlsbad, CA, USA) with stainless steel beads (5 mm; Qiagen, Mississauga, ON, Canada) using a TissueLyser (Qiagen), and subjected to RNA extraction following manufacturers’ instructions. Liver samples were subjected to the homogenization and extraction procedure followed by a second extraction using the phenol-chloroform phase separation method as described previously (28). The total RNA of 40 µg was DNase (6.8 Kunitz units; RNase-Free DNase Set, Qiagen)-treated and column-purified using RNeasy Mini Kit (Qiagen) following the manufacturer’s protocols. RNA integrity and purity were evaluated using 1% agarose gel electrophoresis and NanoDrop spectrophotometry (Thermo Fisher, Mississauga, ON, Canada), respectively. All the column-purified RNA samples showed high integrity and purity (i.e., A260/230 and A260/280 ratios >1.8; and tight 18S and 28S ribosomal RNA bands).

### 2.4 Sample Selection Using qPCR

We selected 12 immune, tissue damage, and wound healing biomarkers (**Table 2**; named as preliminary qPCR in **Figures 7**, **8**) for a preliminary qPCR study on the gill RNA samples (i.e., not on the liver) to compare damaged groups (i.e., GS1, GS2, and GS3) with the pristine gill group (i.e., GS0). Those biomarkers (i.e., *mmp19*, *mmp13b*, *gpx2*, *hif1aa*, *hsp70*, *il1b*, *c1qtn3*, *mucin5ac*, *ladderlectin-like*, *sdhb*, *ctsd*, and *hceb*) were selected based on our group’s experience and relevant literature (8, 29, 32, 33). All the RNA samples were included in this preliminary qPCR. The obtained qPCR data were analyzed *via* linear discriminant analysis (LDA) to select the gill damage group to include in the microarray study (together with GS0) and the most representative biological replicates within each group (i.e., closely clustered in the multivariate space; **Supplementary Figure 3**). Moderately damaged gill (i.e., GS2) was selected as the gill damage group for the microarray analysis. The preliminary qPCR results for the targeted biomarkers relevant to gill remodeling and wound healing and immune and stress response are described in section *Sample Selection for the Microarray Experiment*. The preliminary qPCR for the sample selection, was performed as described in section *Real-Time Quantitative Polymerase Chain Reaction (qPCR) Analysis for the Gill*. The experimental design of the current study is summarized in a schematic diagram (**Supplementary Figure 4**).

**Table 2:** Primers used in the gill either in the preliminary qPCR or the microarray validation experiment, including comparison between microarray Rank Products and qPCR fold-change results for Atlantic salmon transcripts responsive in moderately damaged gill.

Probe identifier^a^	Gene description^b^	Symbol^c^	GenBank accession number	FC: (GS2/GS0) RP^d^	FC: (GS2/GS0) qPCR^e^		Primer sequence 5′–3′	Amplification efficiency (%)	Amplicon size (bp)	Source	
**Gill remodeling and wound healing**											
preliminary qPCR	collagenase 3-like/matrix metalloproteinase-13	*mmp13b*	BT058668.1	–	0.66	F	CTATAGTGGCTCCTTCATGTTTGAG	93.0	116	(29)	
						R	CTTTAAACGGCTCATGAGGGTC				
	matrix metalloproteinase-19	*mmp19*	XM_014132587	–	0.92	F	CTGAACGCAGCCGTTTACT	93.1	132	(29)	
						R	AATATTAGGTGGGAGGCGTTTG				
C077R068	ABI family, member 3 [NESH] binding protein	*abi3bp*	XM_014174255.1	4.3	2.1	F	GCATAAGGATATGCTTTGTTGTGCT	84.6	123	(f)	
						R	GGTCACTTGCAATGAGCTTAGGA				
C101R031	actin, alpha skeletal muscle 2-like	*acta2*	XM_014131025.1	4.2	2.5	F	AGAAGAGCTACGAGCTTCCC	98.5	85	(f)	
						R	AGCGGACTCCATACCAATGA				
C091R125	actin, alpha cardiac-like	*actc1*	XM_014210475.1	2.7	1.6	F	TTCCAGGCCATGTGGATCA	85.3	94	(f)	
						R	GGTGAAGAGGGTAGACTGTTTAGA				
C178R027	aldolase a, fructose-bisphosphate 1	*aldoa*	XM_014159239.1	2.7	6.2	F	CTCCTAACCCTCCCACCATCT	87.2	99	(f)	
						R	TCGAGTAGAGACGGCTTCTTAGG				
C122R160	bone gamma-carboxyglutamate protein	*bglap*	NM_001136551.2	2.3	2.5	F	GAACCAACAGCAAAGAGAAAGATG	91.9	93	(f)	
						R	GGTAGAGGAGTCTCCAATAGAGTG				
C134R091	calsequestrin-2-like	*casq2*	XM_014164852.1	2.1	5.0	F	GAACTACCAGAAGGCCATGAAG	85.2	114	(f)	
						R	TCCAGCACCATCTCAGTCAT				
C002R150	chromatin target of PRMT1 protein-like	*chtop*	XM_014141601.1	0.1	0.9	F	GTTGGGCCTATGAAGAGAACTG	87.2	89	(f)	
						R	GTGTCTACGTTGCACTGATACC				
C254R106	coatomer subunit zeta-1	*copz1*	XR_001329207.1	3.9	0.8	F	ACGCCAAATAGGAAGATAGGCTAAT	103.7	92	(f)	
						R	TAGCTTGACAACAGGCAGACTC				
C236R013	desmin-like	*des*	XM_014183249.1	2.8	10.8	F	TGCTGCCTCATCATATTCCACTATT	109.3	112	(f)	
						R	GCGGGTTTCGATGGTCTTGAT				
C249R072	elastin-like	*eln*	XM_014162760.1	3.9	1.4	F	CGTGCGGTGGGATAGTATTCAT	97.8	107	(f)	
						R	GGATTACCGGGAACAAGTCACA				
C073R110	kelch-like protein 41b	*klhl41b*	XM_014173228.1	2.3	2.9	F	ACCGTGTCAAGACGCTAGAA	93.2	110	(f)	
						R	AGGGTCAGCCTTGATGATGT				
C181R085	myoglobin (myg)	*mb*	BT057357.1	3.1	7.6	F	CTGACTACAACAATCACGGAGGAC	100.7	96	(f)	
						R	CTGCGATGCCTGCGAACTTA				
C092R076	myosin heavy chain, fast skeletal muscle-like	*myh*	XM_014132387.1	5.7	109.0	F	GCCATCTACCTCCGCAAGC	93.0	100	(f)	
						R	ACATCTCTTTGGGCTCAACCAC				
C100R066	myomesin-1-like	*myom1*	XM_014180785.1	2.8	2.2	F	GTCGTGATTATGGCATCCCTTTC	84.2	96	(f)	
						R	CACTGCGTCGTGACAAAGTAA				
C029R004	nebulin	*neb*	XM_014173550.1	2.9	3.0	F	CCGTGTCAGCTCTATTCACTGT	81.6	87	(f)	
						R	CAACTTCAGAACGCACAACTCC				
C223R058	phosphoglycerate mutase 2-1	*pgam2*	NM_001139729.1	2.6	6.5	F	TCCACTCACCCAAACACACAAA	81.6	103	(f)	
						R	TGTCAGACTGTTGTGCCCTTAAC				
C113R032	striatin-interacting protein 2	*strip2*	XM_014152906.1	2.4	1.0	F	TGCAGTGTTTGGATGTTGCT	81.0	103	(f)	
						R	GGACAGTCATTGCACATACCC				
C160R049	troponin T, cardiac muscle isoforms-like	*tnnt2*	XM_014166074.1	5.3	7.4	F	GAGACCATAGGAGTCCATTCCATTC	91.8	82	(f)	
						R	TCACACAAGTCTTCCTTGTCTGTAG				
C148R096	tropomyosin beta chain	*tpm*	XM_014144815.1	4.4	2.9	F	GATCTGGGTTAGGCTTGGTCTTC	91.5	86	(f)	
						R	GCGGTTTCAGGACATTTATTACAGAG				
C103R085	E3 ubiquitin-protein ligase TRIM39-like	*trim39-like*	XM_014153318.1	3.2	0.7	F	TACAGTATGCAGCAGCATTGTAAGA	86.9	115	(f)	
						R	CTTCCACAGAAATGTAACTCCCAGATA				
**Immune and stress-relevant**											
preliminary qPCR	complement C1q tumor necrosis factor-related protein3-like	*c1qtn3*	XM_014134632	–	0.66	F	TGGTCCACACTCACGTCAT	81,79	83		(g)
						R	GACTCCATTTACTGGTGCTGTG				
	cathepsin D	*ctsd*	BT043515	–	0.87	F	AGGACTGTCCATAGAGGAGCA	93.1	142		(g)
						R	GTTGTCAAACGGTGGAGCAAC				
	glutathione peroxidase 2-like	*gpx2*	XR_001319691.1	–	0.60	F	TCATGTACTGTGCTCTCCTGT	103.5	137		(hP)
						R	TGGGCCCAATGCAACTTATG				
	high choriolytic enzyme	*hceb*	XM_014174772.1	–	0.79	F	CCCTGCACAGCTCACAC	86.1	86		(g)
						R	GGCGAGTATTGATTAGAGATCACA				
	hypoxia Inducible Factor 1 Alpha	*hif1aa*	NM_001140022	–	0.75	F	CCCATGTTCACAACAACAGC	87.1	100		(h)
						R	AATGAGAAGGGGCTGAACCT				
	heat shock protein 70	*hsp70*	BT058774	–	0.92	F	GTTATCAATGATTCTACTCGGCC	116.0	148		(h)
						R	CTGCATTGTTGACAGTTTTTCC				
	interleukin 1 beta	*il1b*	AY617117	–	0.64	F	GTATCCCATCACCCCATCAC	98.8	119		(33)
						R	TTGAGCAGGTCCTTGTCCTT				
	*ladderlectin-like*	*ladderlectin-like*	XM_014125761.1	–	0.47	F	AGCAGAGAGTGATCGTCCATGT	88.4	133		(g)
						R	AGACGCCAGGTTTGCTTGAAA				
	mucin5a	*mucin5ac*	JT819124	–	1.15	F	GGGACAGGTGGCGAATTTAT	94.0	97		(g)
						R	TGCTGCCGTCTCCTCTTAT				
	succinate dehydrogenase[ubiquinone] iron-sulfur subunit, mitochondrial-like	*sdhb*	BT125403	–	0.67	F	CTGTGGCCCCATGGTATTAG	90.7	101		(g)
						R	AGGATCCGCAGATACCCTCT				
C139R108	C-X-C motif chemokine receptor 5	*cxcr5*	XM_029773940.1	2.5	3.0	F	CACCGCCACAACCACTAAA	85.8	116		(f)
						R	AGATCAGATTTAACCGTCAGTATCC				
C011R100	DNA damage-inducible transcript 4 protein-like	*ddit4*	XM_014155366.1	0.5	1.1	F	TGCTGAAAGAAACCAGAGACTT	92.5	83		(f)
						R	TGATGAGCGTTGTAGGTAGGA				
C006R072	gamma-aminobutyric acid receptor subunit alpha-2-like	*gabra2*	XM_014210615.1	0.4	0.8	F	GTGACAGAGGTGAAGACTGATATTT	83.9	111		(f)
						R	CCGTTCGTCAATCCAACTCTG				
C266R066	Ig kappa chain V-III region MOPC 63	*igkv3*	BT046734.1	5.3	3.0	F	GGCCATCAGTGTCTATCCTGGTA	89.6	91		(f)
						R	GAGTCCCAGAATGGAGTTTGTTCC				
C123R073	olfactomedin-4-like	*olfm4*	XM_014124361.1	2.4	1.2	F	TGTGCCGCTATGACCTACAA	91.4	114		(f)
						R	CGGTGTCCGTCCAGTCTTTA				
C010R062	snaclec 1-like	*snaclec 1-like*	XM_014158126.1	0.3	0.1	F	CTGAATGATCTGGAGCAGGAAGG	87.1	200		(f)
						R	AAGAGTTCAGTGCCACACAGTC				
C038R122	UDP-glucuronosyltransferase 2C1	*ugt2c1*	XR_001324390.1	0.4	1.5	F	GCACAGCTTCCTCGTGATTT	86.1	96		(f)
						R	AGCTGGTCTGTCTCCTTTGT				
**Normalizers**											
	elongation factor 1 alpha-1	*ef1a1*	AF321836	–	–	F	TGGCACTTTCACTGCTCAAG	91.5	197		(30)
						R	CAACAATAGCAGCGTCTCCA				
	polyadenylate-binding protein 1	*pabpc1*	EG908498	–	–	F	TGACCGTCTCGGGTTTTTAG	90.2	108		(31)
						R	CCAAGGTGGATGAAGCTGTT				

a Probe’s identifier of the 44 K array detected by Rank Products (RP) analysis and targeted by qPCR for validation.

b Name/s or alias obtained from annotation.

c Official gene symbols based on multiple annotations; majority are represented in HGNC (https://www.genenames.org/) and/or GeneCards (https://www.genecards.org/) databases.

d Fold-change (FC) for RP, The FC was calculated using the Bioconductor package, RankProd.

e FC for the qPCR (i.e., GS2/GS0).

f Microarray identified novel biomarker; primers are designed as described in section Real-time Quantitative Polymerase Chain Reaction (qPCR) Analysis for the Gill.

g Primers were designed based on studies on references (8, 32, 33).

h Primers were designed as part of two Genomic Applications Partnership Program projects [GAPP # 6604: Biomarker Platform for Commercial Aquaculture Feed Development project; and GAPP #6607: Integrated Pathogen Management of Co-infection in Atlantic salmon (IPMC) project] funded by the Government of Canada through Genome Canada and Genome Atlantic, and EWOS Innovation, now part of Cargill, Incorporated (to MLR). The IPMC project was also funded by the Government of Newfoundland and Labrador through the Department of Tourism, Culture, Industry and Innovation (Leverage R&D award #5401-1019-108). Those primers were designed by Dr. Jennifer R. Hall and Xi Xue.

### 2.5 Microarray Hybridization and Data Acquisition

The gill total RNA of five individuals from each GS0 and GS2 were included in the microarray analysis using a common reference design (**Figure 1B**). The microarray experiment was designed and performed according to the MIAME guidelines (34). These analyses were carried out using the consortium for Genomic Research on All Salmonids Project (cGRASP)-designed Agilent 44K salmonid oligonucleotide microarray (25) as described in Xue et al. (28). Briefly, anti-sense amplified RNA (aRNA) was *in vitro* transcribed from 1 µg RNA of each sample using the Amino Allyl MessageAmp II aRNA Amplification kit (Ambion/Life Technologies), following the manufacturer’s instructions. The aRNA quality and quantity evaluations were assessed using 1% agarose gel electrophoresis and NanoDrop spectrophotometry (Thermo Fisher, Mississauga, ON, Canada), respectively. From each sample used in the experiment, 5 µg of aRNA were collected to generate a common reference pool (**Figure 1B**). Following a standard molecular procedure, 20 µg of aRNA were precipitated overnight for each individual and resuspended in 9 µl coupling buffer (Ambion/Life Technologies). The aRNA pool was then divided into three aliquots and precipitated and resuspended following the same approach as the individual samples. The aRNA was labeled with Cy3 fluor (GE HealthCare, Mississauga, ON, Canada) for the common reference pool and Cy5 fluor for the experimental individuals, following the manufacturer’s instructions. The labeling efficiency for the dye was measured with the “microarray” function of the NanoDrop spectrophotometer. Equal quantities (825 ng) of each labeled aRNA from one experimental sample and the common reference were pooled, fragmented following the manufacturer’s instructions, then co-hybridized to an Agilent 44K salmonid oligonucleotide microarray (GEO accession # GPL11299) (28) following the manufacturer’s instructions (Agilent, Mississauga, ON, Canada). The arrays were hybridized at 65°C for 17 h at 10 rpm using an Agilent hybridization oven. After hybridization, the array slides were immediately washed as recommended by the manufacturer. Each microarray slide was scanned at 5 µm resolution using a SureScan D Microarray Scanner (G2600D, Agilent Technologies) using Agilent Scan Control Software (v9.1.11.7, Agilent Technologies) by applying a built-in protocol (Agilent_HD_GX_2color). The photomultiplier tube sensitivity for Cy3 and Cy5 dye channels were adjusted at 100%. The signal intensity data were extracted and Loess-normalized using Agilent Feature Extraction Software v12.0 (Agilent).

### 2.6 Microarray Data Analysis

The microarray data were processed using GeneSpring v14.9 (Agilent). Probes with low or marginal quality and absent values in more than 25% of all 10 arrays were removed from the dataset, and the missing values were imputed. Rank Products (RP), a non-parametric statistical method (35) was used to determine the differentially expressed probes (DEPs), as this method is less sensitive to high biological variability within groups than Significance Analysis of Microarrays (SAM) (36–38). The RP was performed at 5% percentage of false-positives (PFP) using the Bioconductor package, RankProd (35). The DEPs were annotated using the contiguous sequences (contigs) from which the 60mer oligonucleotide probes on the array were designed against the Swiss-Prot database (April 2019 version). The BLASTx searches of NCBI’s non-redundant (nr) amino acid sequence and Swiss-Prot databases (E-value < 1e-05) were performed using Blast2GO software (BioBam Bioinformatics S.L., Valencia, Spain) (39, 40). Then, each DEP annotation was manually confirmed using the best BLASTn and BLASTx hit with E-value < 1e-05. HUGO Gene Nomenclature Committee (HGNC; https://www.genenames.org/) and/or GeneCards (https://www.genecards.org/) databases were used to assign human gene symbols. The 44K redundancy (i.e., multiple probes targeting the same gene) was accounted for, to reduce the DEP list to a list of differentially expressed genes (DEGs). The DEG list was then used for Gene Ontology (GO) term enrichment analysis. Over-represented biological process (BP), molecular function (MF), and cellular component (CC) GO terms were identified through right-sided hypergeometric tests using the human GO database (UniProt: 27.02.2019), with a Benjamini-Hochberg method-corrected p-value threshold of 0.05. This analysis was carried out using ClueGO (41) plugin in Cytoscape (v3.5.1), which allowed GO term interconnection/clustering based on kappa statistics. The kappa coefficient threshold for the analysis was 0.4.

### 2.7 Real-Time Quantitative Polymerase Chain Reaction (qPCR) Analysis for the Gill

Twenty-seven microarray-identified genes were selected for the microarray confirmation using qPCR (**Figures 7**, **8**; named as microarray-identified biomarkers). As mentioned in section *Sample Selection Using qPCR*, another 12 genes (**Figures 7**, **8**; named preliminary qPCR) were used for the preliminary qPCR on the gill RNA, and used for sample choice. The following procedure was used for both the preliminary qPCR (see Section *Sample Selection Using qPCR*) and the qPCR confirmation of microarray results. Both the preliminary qPCR and validation qPCR were performed using all sampled fish (i.e. all the RNA samples). The first-strand cDNA templates for qPCR were synthesized following the manufacturer’s instructions, using 1 µg of DNase-treated and column-purified total RNA, random primers (250 ng; Invitrogen/Life Technologies), dNTPs (0.5 mM final concentration; Invitrogen/Life Technologies), and M-MLV reverse transcriptase (200 U; Invitrogen/Life Technologies) in 1 X first strand buffer and DTT (10 mM final concentration) at 37°C for 50 min. The qPCR amplifications were performed in 13 µl reaction volumes containing 1× Power SYBR Green PCR Master Mix (Applied Biosystems/Life Technologies), 50 nM of both the forward and reverse primers and 4 µl of cDNA [corresponding to 5 ng input total RNA; dilution performed using DNase/RNase-free distilled water (Invitrogen/Life Technologies)]. The qPCR analysis program consisted of 1 cycle of 50°C for 2 min, 1 cycle of 95°C for 10 min, and 40 cycles of 95°C for 15 s and 60°C for 1 min with fluorescence detection at the end of each 60°C steps. The qPCR reactions were performed in triplicates, no-template controls were included and the dissociation curve analysis was performed for each plate.

All primers in the current study used for the gill are presented in **Table 2**. Primers were designed using the PrimerQuest design tool (www.idtdna.com/Primerquest/Home/Index) or adopted from previous studies (29, 33). Primer quality checks were performed as previously described (42). Primer pairs passing quality testing showed amplification of a single product and no signs of primer-dimers (dissociation curve analysis). All the amplification efficiencies were tested using equal quantity of input total RNA of the gill cDNA pools from individuals designated in GS0 (n=14; pool 1) and GS2 (n=10; pool 2). Average amplification efficiencies from the two pools' standard curves are reported (**Table 2**). The standard curves were generated using 1:3-fold serial dilution and using 5 points starting with cDNA synthesized from 10 ng of total RNA input.

Five normalizers *polr2a* (*RNA polymerase II subunit A*), *rpl32* (*60S ribosomal protein*), *ef1a* (*elongation factor 1α*), *pabpc1* (*polyadenylate-binding protein 1*), and *eif3d* (*eukaryotic translation initiation factor 3 subunit D*) were tested on the gill cDNA. The Ct values of all individuals (i.e., for the gill: n=14 GS0, n=11 GS1, n=10 GS2, n=9 GS3) for all candidate normalizer genes were analyzed using geNorm (qBASE plus, Biogazelle NV, Belgium) (43). *Pabpc1* and *ef1a1* were determined as the best normalizers with M-values of 0.21 and 0.25, respectively.

The relative quantities (RQs) of all genes of interest (GOIs) were calculated for each gill score using all the gill samples (n=44). The RQs were determined using the qBase relative quantification framework (44, 45). This was performed by using the Ct values measured for GOIs, with normalization to both *pabpc1* and *ef1a1* for the gill tissue, and with the amplification efficiencies incorporated. The sample with the lowest normalized expression was used as an internal calibrator for each GOI (i.e., RQ value= 1.0). The RQs values are presented as mean ± SE.

### 2.8 qPCR Analysis for the Liver

Twenty-one biomarkers involved in APR and other damage-relevant biological processes (e.g., wound healing, immunity, transcription factors and stress relevant biomarkers) were targeted in the liver qPCR (**Table 3**). Those biomarkers were chosen based on the identified dysregulated pathways in the gill tissue (overlapping biomarkers were *chtop*, *strip2*, *igkv3*, *olfm4*, *ugt2c1*, and *ddit4*; **Table 3**) and other possible systemic dysregulated pathways (e.g., APR). All the steps for the qPCR were performed following the described methods in section 2.7. All the amplification efficiencies were tested using liver cDNA pools from individuals designated in GS0 (n=32; pool 1) and GS2 (n=10 pool 2) (**Table 3**). Average amplification efficiencies from the two pools' standard curves are reported (**Table 3**). Also, five normalizers (same used in the gill study; mentioned above in section *Microarray Data Analysis*) were tested on the liver cDNA. The Ct values of all individuals (i.e., for the liver: n=32 GS0, n=9 GS1, n= 10 GS2, n=7 GS3) and all candidate normalizers were analyzed using geNorm (qBASE plus, Biogazelle NV, Belgium) (43). *Eif3d* and *rpl32* were determined as the best normalizers with an M-value of 0.41 for both. Primers used for the liver qPCR, either newly designed or adopted from previous studies (28, 42, 46), are presented in **Table 3**. All the steps for the qPCR were performed following the described methods in section *qPCR Analysis for the Gill*. The Ct values of all 58 samples of the liver were used to calculate the RQs of all GOIs following the same method described in section *Real-time Quantitative Polymerase Chain Reaction (qPCR) Analysis for the Gill*.

**Table 3:** Primers used in the liver tissue qPCR across all gill scores of Atlantic salmon.

BLASTx identification/gene name^a^	Symbol	Accession number		Sequence 5′–3′	Efficiency (%)	Size (bp)	Source
**Wound healing**							
Chromatin target of PRMT1 protein-like	*chtop*	XM_014141601.1	F	GTTGGGCCTATGAAGAGAACTG	102.0	89	b
			R	GTGTCTACGTTGCACTGATACC			
Striatin-interacting protein 2	*strip2*	XM_014152906.1	F	TGCAGTGTTTGGATGTTGCT	107.0	103	b
			R	GGACAGTCATTGCACATACCC			
**Apoptosis**							
Bcl2/adenovirus e1b 19 kda protein- interacting protein 3-like	*bnip3l*	BT058694.1	F	TCAGTCACCCAGCATCTCTG	90.8	113	c
			R	ATCAACTGTCCTGCCCTGAC			
Apoptosis-inducing factor 2	*aifm2*	XM_014154057.1	F	ACATGGTGGCCTCCTATCAG	102.0	120	c
			R	CTGCAGCCATCTCTACACCA			
Cathepsin D	*ctsd*	BT043515	F	AGGACTGTCCATAGAGGAGCA	95.0	142	b
			R	GTTGTCAAACGGTGGAGCAAC			
**Blood coagulation**							
Prothrombin	*f2*	EG773276.1	F	GGCTTCAAACCAGAGGAACA	103.0	137	c
			R	TCCCTGTCACATCCTTCTCC			
Heparin cofactor II (Serpin D1)	*serpind1b*	BI468058.1	F	ACATGCGCAGCTTTACCAG	98.0	115	c
			R	TCGGAAGAGTCTGTGCGTAA			
Hemopexin-like	*hpx*	CK896897/XM_014174610.1	F	GTGGATGCCGTCTTCTCCTA	96.9	125	c
			R	AGCACCTCCTTCAAGGGTTT			
**Inflammation-associated**							
C-reactive protein	*crp*	BT058269	F	TCTCTAGCAACCCCCTCTGA	97.0	149	c
			R	TCCCACGTGACACAAAAAGA			
Leukocyte cell derived chemotaxin 2	*lect2a*	BT059281	F	AAGGCTTTACCATGAGGACTGC	107.0	80	(30)
			R	CTTGACCATCTCGCACTCTGAC			
**Immunity**							
Ig kappa chain V-III region MOPC 63	*igkv3*	BT046734.1	F	GGCCATCAGTGTCTATCCTGGTA	88.4	91	b
			R	GAGTCCCAGAATGGAGTTTGTTCC			
Olfactomedin-4-like	*olfm4*	XM_014124361.1	F	TGTGCCGCTATGACCTACAA	88.7	114	b
			R	CGGTGTCCGTCCAGTCTTTA			
UDP-glucuronosyltransferase 2C1	*ugt2c1*	XR_001324390.1	F	GCACAGCTTCCTCGTGATTT	98.0	96	b
			R	AGCTGGTCTGTCTCCTTTGT			
DNA damage-inducible transcript 4 protein-like	*ddit4*	XM_014155366.1	F	TGCTGAAAGAAACCAGAGACTT	100.3	83	b
			R	TGATGAGCGTTGTAGGTAGGA			
Cathelicidin	*campa*	GQ870278.1	F	AAGCCAGAAAATGCTCCAGA	111.0	107	c
			R	ACCCTCAGGACGACCAATTA			
**Transcription factors**							
Peroxisome proliferator-activated receptor gamma	*pparg*	NM_001123546	F	GAGGCCGTACAAGAGGTCAC	89.9	107	(46)
			R	ATGACCTCGATGACCCCATA			
Peroxisome proliferator-activated receptor beta	*pparb1a*	NM_001123635	F	CAGCTGATCAACGGTACGAC	84.5	112	(46)
			R	TGCTCTTGGCAAACTCAGTG			
**Stress relevant biomarkers**							
Cytochrome P450 3A27 B	*cyp3a27*	BT056998	F	GCTGTTTGATGCATTGTCCTT	107.0	135	c
			R	TTCAGCAGGTTAGCAGAGTGCC			
Hypoxia inducible factor 1 alpha	*hif1aa*	NM_001140022	F	CCCATGTTCACAACAACAGC	95.0	100	b
			R	AATGAGAAGGGGCTGAACCT			
Heat shock protein 70	*hsp70*	BT058774	F	GTTATCAATGATTCTACTCGGCC	85.0	148	b
			R	CTGCATTGTTGACAGTTTTTCC			
Glutathione peroxidase 3	*gpx3*	BT072794	F	CTGTGGTTGTGTCCCAAATG	88.8	86	c
			R	CGCAAATGACACCCTATTCC			
**Normalizers**							
Eukaryotic translation initiation factor 3 subunit D	*eif3d*	GE777139	F	CTCCTCCTCCTCGTCCTCTT	94.4	105	(42)
			R	GACCCCAACAAGCAAGTGAT			
60S ribosomal protein L32	*rpl32*	BT043656	F	AGGCGGTTTAAGGGTCAGAT	92.0	119	(28)
			R	TCGAGCTCCTTGATGTTGTG			

a Official gene symbols based on multiple annotations; majority are represented in HGNC (https://www.genenames.org/) and/or GeneCards (https://www.genecards.org/) databases.

b Same Primers used in the gill qPCR.

c Primers were designed as part of two Genomic Applications Partnership Program projects [GAPP # 6604: Biomarker Platform for Commercial Aquaculture Feed Development project; and GAPP #6607: Integrated Pathogen Management of Co-infection in Atlantic salmon (IPMC) project] funded by the Government of Canada through Genome Canada and Genome Atlantic, and EWOS Innovation, now part of Cargill, Incorporated (to MLR). The IPMC project was also funded by the Government of Newfoundland and Labrador through the Department of Tourism, Culture, Industry and Innovation (Leverage R&D award #5401-1019-108). Those primers were designed by Dr. Jennifer R. Hall.

### 2.9 Statistical Analyses

Transcript expression differences between specified gill scores in the gill and liver were analyzed using one-way ANOVA, followed by Tukey’s post-hoc test for pairwise comparisons between gill score groups (i.e., GS0, GS1, GS2, GS3) using SPSS (IBM SPSS Statistics, Version 25, Armonk, NY, USA). All residuals were examined for normality and homoscedasticity (i.e., Shapiro–Wilk and Levene’s tests, respectively) for each gene separately. Furthermore, QQ-plots were generated to check the data normality. All the reported data fulfilled the assumptions of normality and homoscedasticity. To compare the statistical difference between GS0 and GS2, a Student’s t-test was performed for both gill and liver qPCR studies. Due to the observed gradient responses (e.g., *campa* and *pparb1a*) of the targeted transcripts in the liver tissue, we performed another Student’s t-test to compare GS0 and GS3 (i.e., only for the liver qPCR study). The fold-change (FC) from the microarray log_2_ ratios and the log_2_ FC of qPCR RQs were analyzed using linear regression to validate the microarray results (28–30). The significance level was designated as p-value ≤ 0.05; however, GOIs displaying p-values between 0.05 and 0.10 (i.e., response trend in the gill either using one-way ANOVA or Student’s t-test) were also considered for multivariate analyses and Pearson’s correlation analyses of the gill qPCR study. LDA was the multivariate analysis used on the preliminary qPCR data for gill score groups and biological replicate selection for the microarray analysis (i.e., samples closely clustered were selected for the microarray; **Supplementary Figure 3**). Principal component analysis (PCA) was used for the identification of expression patterns among the GOIs selected for the gill qPCR (i.e., transcripts with p-value ≤ 0.1) and liver qPCR studies (i.e., all targeted transcripts). The LDA was conducted using the “MASS” package (47, 48) in the R environment. The PCA was performed using the procomp function and plotted using “ggbiplot” R package (49). PC1 and PC2 scores were analyzed *via* one-way ANOVA and Tukey’s post-hoc test for differences among gill score groups. Also, the contribution of the top 10 genes to PC1 and PC2 was plotted. Correlations among GOIs and between GOIs and fish performance parameters (e.g., fish weight, condition factor) were analyzed using “ggcorr()” function of the R package GGally.

## 3 Results

### 3.1 Animals, Environmental Stress-Relevant Data and Mortalities

**Figure 2:**
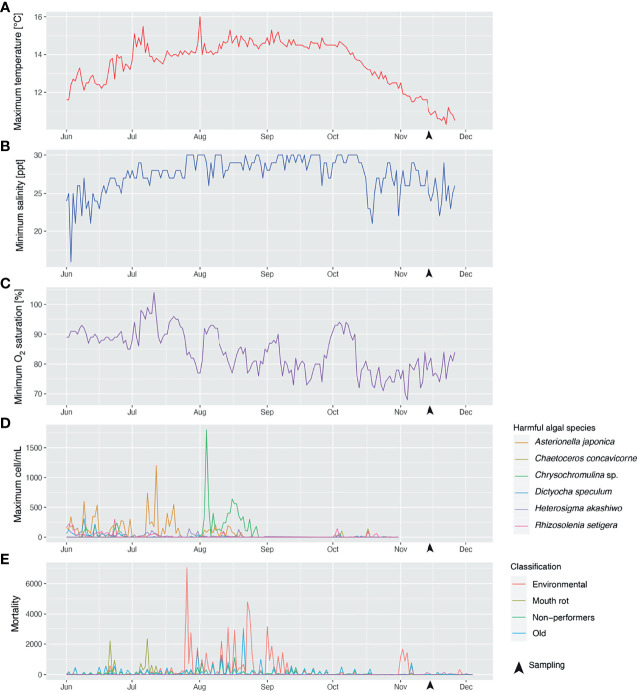
Time series of maximum water temperature **(A)**, minimum salinity **(B)**, minimum oxygen saturation **(C)** recorded at the farm site during the period June 1–November 26, 2017; concentration (cells/mL) **(D)** of various phytoplanktonic algae species potentially harmful for farmed Atlantic salmon, recorded at the farm site from June 1–October 26, 2017; and fish mortality **(E)** recorded at the farm site during the period June 1–December 4, 2017. Fish mortalities were classified into different categories depending on the putative cause of death; i.e., mortalities attributed to environmental stress (e.g., algal blooms, hypoxia events) were classified as “environmental”; mortalities suspected to be caused by *Tenacibaculum maritimum* infection [causative agent of ulcerative stomatitis (also known as mouth rot disease) in salmonids; note: infection was not analytically confirmed] were annotated as “Mouth rot”; salmon euthanized due to their poor growth performance were designated as “non-performers”; fish carcasses too deteriorated to be classified were named “old”.

The average weight (with standard deviation) for all of the collected fish in November 2017 was 1348 ± 398 grams. **Figure 2** shows the time series of the water temperature, salinity, and oxygen saturation values that could pose a threat to Atlantic salmon production, i.e., the daily maximum water temperature (**Figure 2A**) and the minimum salinity (**Figure 2B**) and oxygen saturation (**Figure 2C**). Maximum water temperatures did not reach values above the range considered optimal for Atlantic salmon growth (16-18°C; based on what is mentioned in (50)) from June 1 to November 26, 2017. According to the data provided by the farm, the daily maximum water temperature increased from June (~12°C) to August (~14.5°C) and did not start to decrease until early October. The highest maximum water temperature, 16°C, was recorded on August 1^st^ at 1 m depth, and the maximum water temperature on the sampling dates (i.e., November 13-14) was 11-11.6°C. Fluctuations in minimum salinity values showed more abrupt changes from June 1 to November 26; e.g., on June 2-3, the minimum salinity decreased from 25 ppt to 16 ppt (56% reduction). The daily minimum salinity also appeared to increase from June to August and to increase from October onwards; however, the trend was less evident than with the maximum water temperature. The daily minimum oxygen saturation showed a progressive decrease from June (85-90%) to August (75-80%). Minimum oxygen saturation levels that could be considered moderate hypoxia [i.e., 70%; reduced growth of Atlantic salmon was previously reported with 70% oxygen saturation (51)] were recorded on November 3-4. At the sampling dates (i.e., November 13-14), the minimum oxygen saturation was 78-80%. **Figure 2D** shows the time series of the maximum cell concentration of 6 known harmful phytoplanktonic algae detected at the farm site at levels higher than 100 cell/mL for the period June 1-October 30. Most instances in which harmful algal species were identified were between June and September. Five episodes with maximum *Asterionella japonica* cell concentrations > 500 cell/mL (1,200 cell/mL on July 12) were recorded during this period. *Chrysochromulina* sp. cell concentrations were > 100 cell/mL for most days in August, with two episodes of significance: one on August 4, when cell concentration reached 1,800 cell/mL; and another on August 16-18, a period during which cell concentration stayed > 500 cell/mL. Maximum cell concentrations < 150 cell/mL were detected on different dates in October (~one month before the sampling date) for *Rhizosolenia setigera*, *Dictyocha speculum*, *Chaetoceros concavicorne*, *Asterionella japonica*, and *Heterosigma akashiwo* (ordered from the most frequently detected/highest maximum cell concentration to the least frequently detected/lowest maximum cell concentration). **Figure 2E** corresponds to the time series of the fish mortality for the period June 1-December 4. The most significant mortality events (> 2,000 dead fish) were 2 classified as “Mouth rot” [i.e., fish showed clinical signs compatible with ulcerative stomatitis (alias mouth rot disease)] occurring on June 21 and July 8; 8 classified as “environmental” [i.e., putatively caused by environmental stressors (e.g., harmful algae)], scattered throughout late July and early September; and one event classified as “old” (i.e., the cause of death could not be assessed due to the deterioration of the fish carcass) on July 21. The closest mortality events to the sampling point, with 1000-1700 dead fish, were classified as "environmental" and occurred on November 1, 2 and 4.

### 3.2 Sample Selection for the Microarray Experiment

The majority of GS0 individuals were loaded negatively on LD1, while most of GS2 individuals were loaded positively on LD1, showing delineation between the biomarker transcript expression responses of the fish in these two groups (**Supplementary Figure 3**). Also, the majority of GS1 individuals were loaded positively on LD1 with some overlapping with GS3 (**Supplementary Figure 3**). While all four gill scores (using all available individuals) were included in the qPCR experiments, we decided to exclude GS1 and GS3 samples from the microarray study and focus on GS0 *vs*. GS2 over concern that: 1) GS1 may be difficult to differentiate from GS0 at the transcriptome level; and 2) there may be high variability in the transcriptomes of heavily damaged gills, with transcript expression signatures of GS3 potentially dominated by cell death (e.g., apoptosis, necrosis). For the microarray experiment, we selected individuals (bold in **Supplementary Figure 3**) that clustered together (within the gill score group) in the LDA and segregated from those of the other groups.

### 3.3 Transcriptomic Changes in Response to Moderate Gill Damage

**Figure 3:**
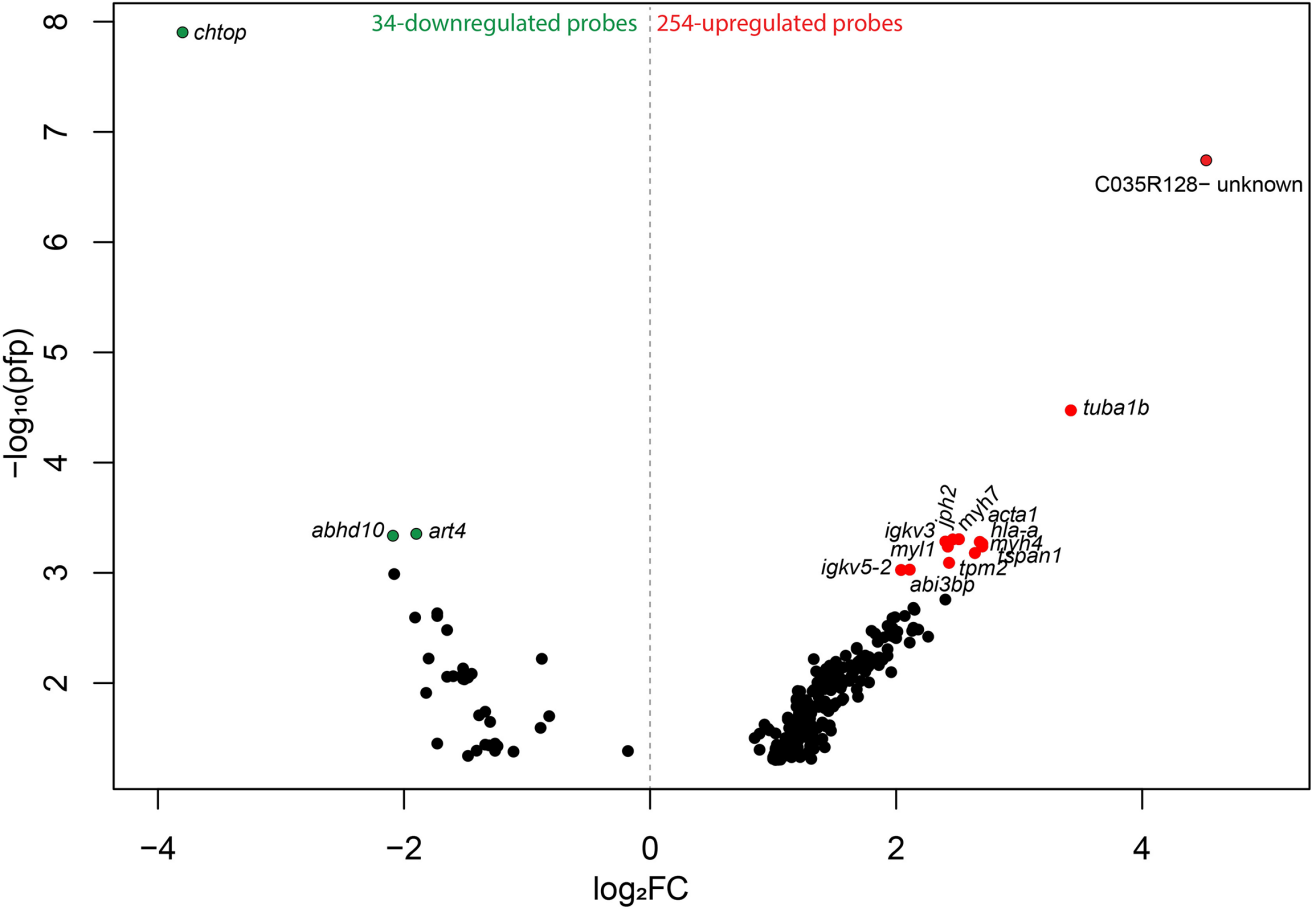
Volcano plot reporting -log_10_ percentage of false-positives (pfp) against Rank Products log_2_ fold-changes (log_2_FC; calculated using the Bioconductor package, RankProd.). The colored dots represent the highest –log_10_ pfp≤0.001 from the upregulated and downregulated probes (red and green, respectively) (**Supplemental File 1**). Probes are labeled with the gene symbols of the putative human orthologues, and one uncharacterized probe is labeled with the probe identifier (ID).

To investigate the global transcript expression response of moderately damaged gill (i.e., GS2), caused by several environmental factors [the most notable likely cause being successive HABs (**Supplementary Figure 1**)], compared with pristine gill (i.e., GS0) subjected to the same environmental conditions, we used a 44K cGRASP salmon microarray platform (25). Using RP statistical analysis, we identified 254 upregulated and 34 downregulated probes in GS2 gills compared with GS0 gills (**Figure 3**). These 288 DEPs, the corresponding appropriate gene symbols, p-values, PFP, and the FC values from the RP, are listed in **Supplementary Table 1**. The 288 DEPs were visualized in a volcano plot showing log_2_-transformed fold-changes (log_2_FC) *vs*. –log_10_(PFP) (**Figure 3**). The lowest PFP from the downregulated probes were *chtop*, *abhd10*, and *art4*, while the lowest PFP from the upregulated probes with gene annotation were *tuba1b*, *tpm2*, *tspan1*, *myh7*, *jph2*, and *igkv3*.

**Figure 4:**
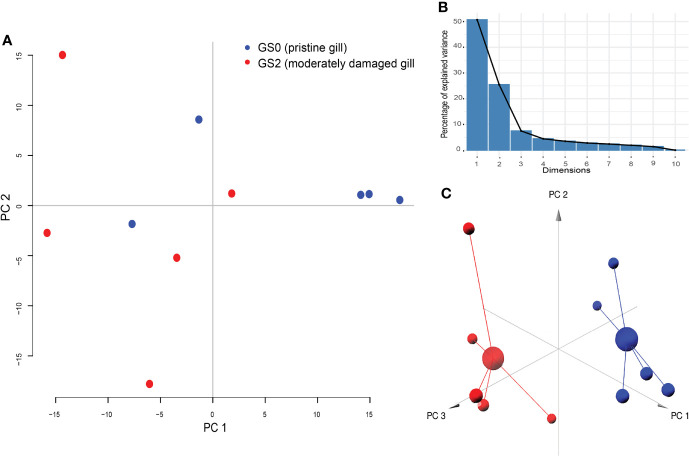
Results of principal component analysis (PCA) plotted on two dimensions (Panel **A**) for the gill differentially expressed microarray log_2_ ratio (Cy5/Cy3). PC1 explained 50.72%, PC2 explained 25.49%, and PC3 explained 7.46% of the variability. Panel **(B)** Bar-plot of the percentage of the explained variance for each PC (dimension). Panel **(C)** PCA plotted on three dimensions for the gill differentially expressed microarray log_2_ ratio (Cy5/Cy3) data.

**Figure 5:**
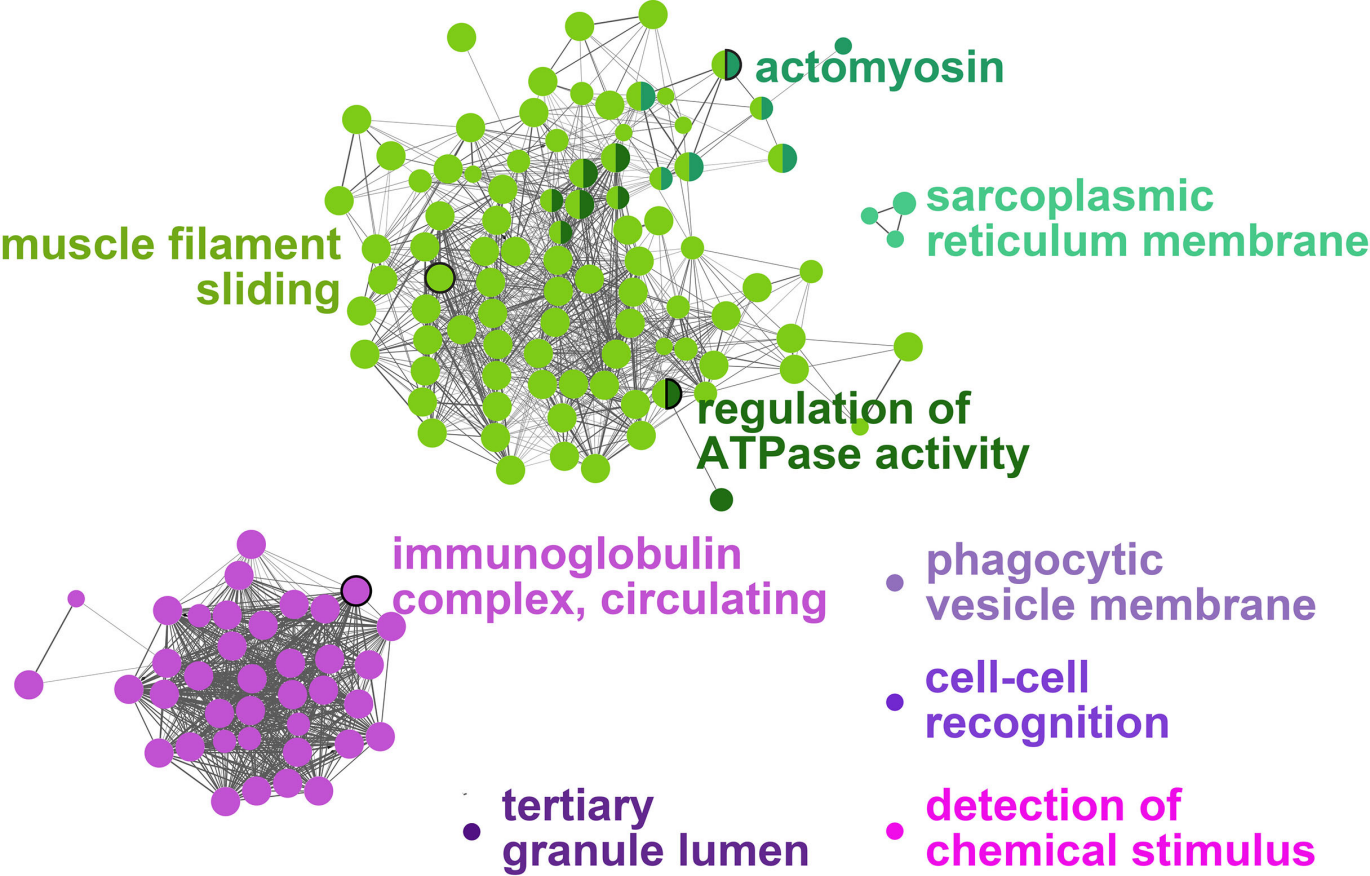
Gene-Ontology (GO) term enrichment and pathway term network analysis of DEGs. The GO-term enrichment analysis was performed using ClueGO plugin in Cytoscape. The p-value was adjusted at 0.05, kappa score level was ≥0.4 on ClueGO and Benjamini-Hochberg correction was used. Biological Process, Cellular Component, and Molecular Function were the selected ontologies on ClueGO. Terms related to gill remodeling and wound healing are colored in different shades of green, while terms related to immunity and stress are colored in different shades of purple. A complete list of the enriched GO terms is found in **Supplementary Table 2**, while the leading GO terms are also labeled in the figure.

All the log_2_ ratio (Cy5/Cy3) data of the identified microarray probes were used to generate a PCA plot (**Figures 4A, C**). Although the PC1 and PC2 did not completely separate the GS0 and GS2 individuals (**Figure 4A**) in the two-dimensional plot, utilizing a 3-dimensional approach (i.e., using PC1, PC2 and PC3 scores) more clearly separated the responses of GS0 and GS2 fish (**Figure 4C**). The percentage of the explained variance for each PC dimension were plotted on **Figure 4B**. Then, all the DEPs (i.e., upregulated, and downregulated) were subjected to GO term enrichment analysis. The analysis defined 148 enriched GO terms (**Supplementary Table 2**) classified into two main themes: gill remodeling and wound healing (green shades; **Figure 5**), or immune and stress-relevant (purple shades; **Figure 5**). The most significant enriched GO terms were actin-myosin filament sliding (GO:0033275) and muscle filament sliding (GO:0030049) (using p-value corrected with Bonferroni step-down ≤ 0.05). Within the immune and stress-relevant theme, adaptive immune response (GO:0002250) was the most significantly enriched GO term.

**Figure 6:**
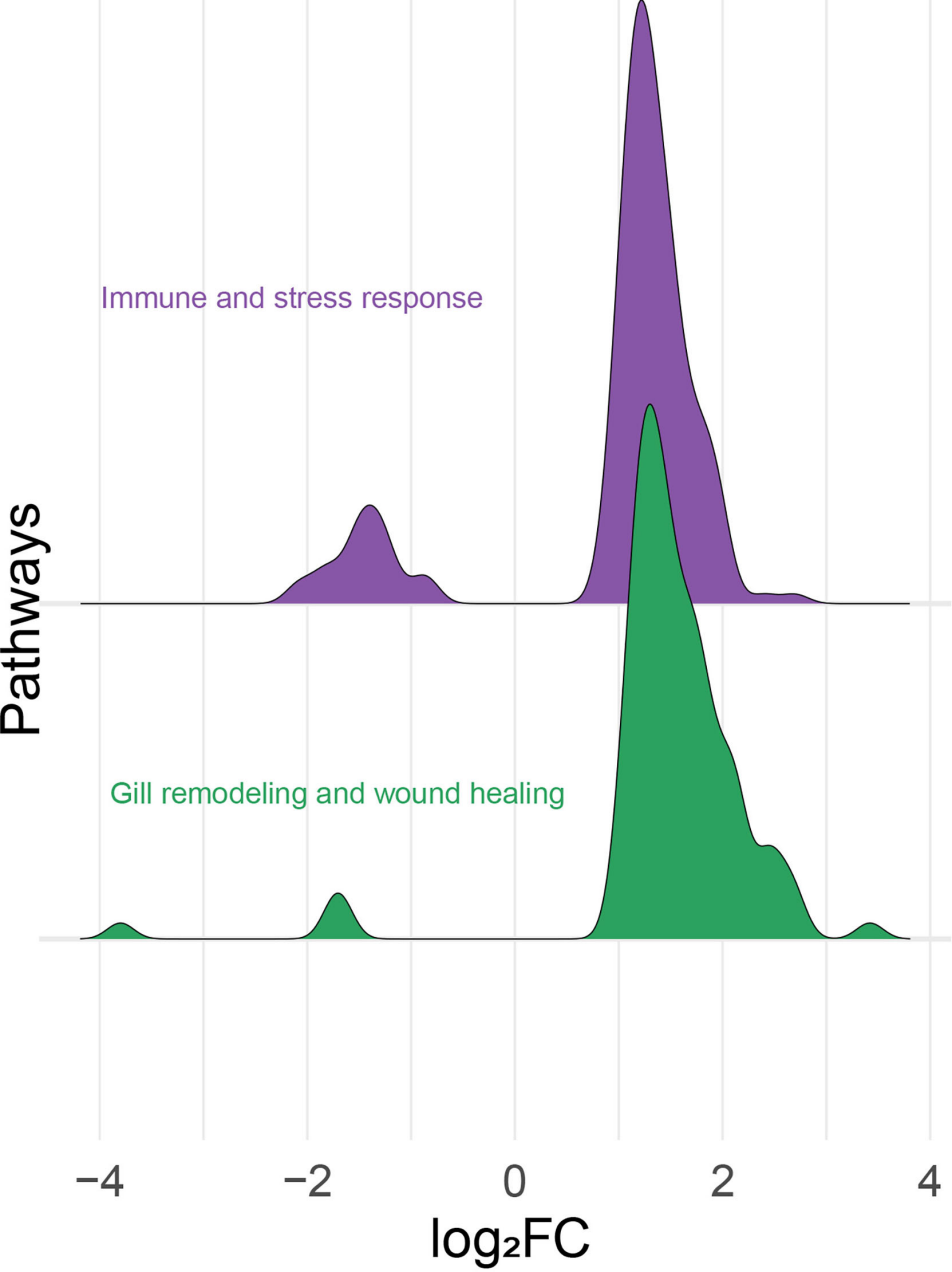
Histogram of the frequency density of log_2_-transformed fold-changes (log_2_FC) for the differentially expressed probes of the “immune- and stress-relevant” and “gill remodeling and wound healing” themes. Purple and green colors indicate the two manually defined themes, immune and stress relevant, and gill remodeling and wound healing, respectively.

All the identified DEPs were classified manually as either immune and stress-relevant (purple; **Figure 6**) or gill remodeling and wound healing-relevant (green; **Figure 6**). The density plot (**Figure 6**) shows the upregulated and the downregulated probes from the two main themes. Both pathways had more upregulated probes than downregulated probes. The majority of the upregulated probes present were with log_2_FC values below 2 (**Figure 6**). Only few probes related to gill remodeling and wound healing were higher than log_2_FC of 3. Also, most of the downregulated probes present were higher than -2 log_2_FC. Few probes related to gill remodeling and wound healing were below log_2_FC of -3.

### 3.4 Preliminary and Confirmation qPCR of Selected Transcripts in the Gill

**Figure 7:**
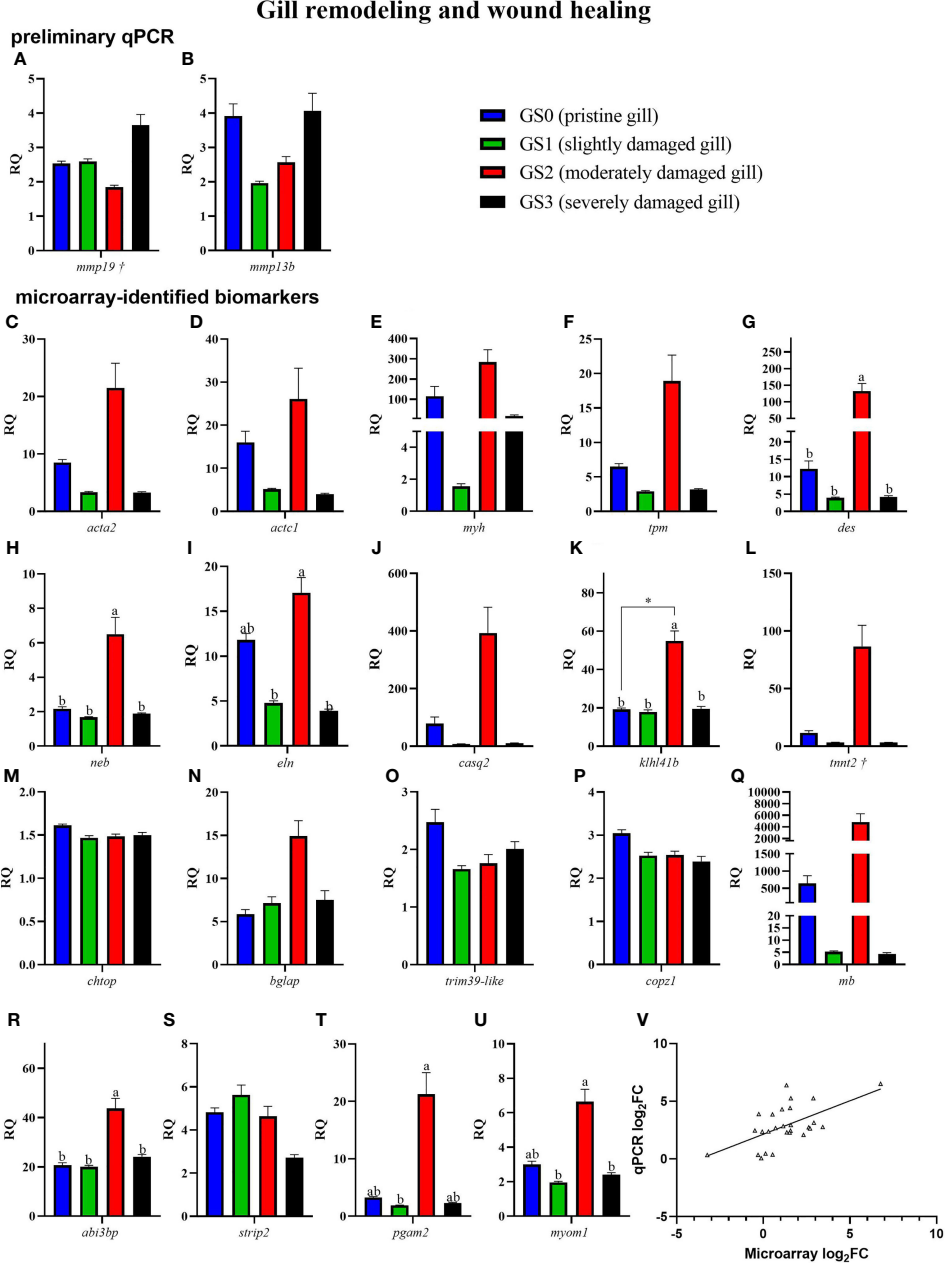
Bar plot of gill transcripts related to gill remodeling and wound healing (panel **A–U**). On the lower right side, Panel **(V)** shows a scatterplot of log_2_ fold-change from Rank Products microarray data *vs*. qPCR log_2_ fold-change. Different letters indicate a significant difference between groups using one-way ANOVA. Asterisk (*) shows significance (p< 0.05) between GS0 and GS2 using t-test. Gene symbols followed by a dagger (†) are associated with p-values between 0.05 and 0.10 using either t-test (GS0 vs GS2; i.e., *mmp19*) or one-way ANOVA (i.e., *tnnt2*).

**Figure 8:**
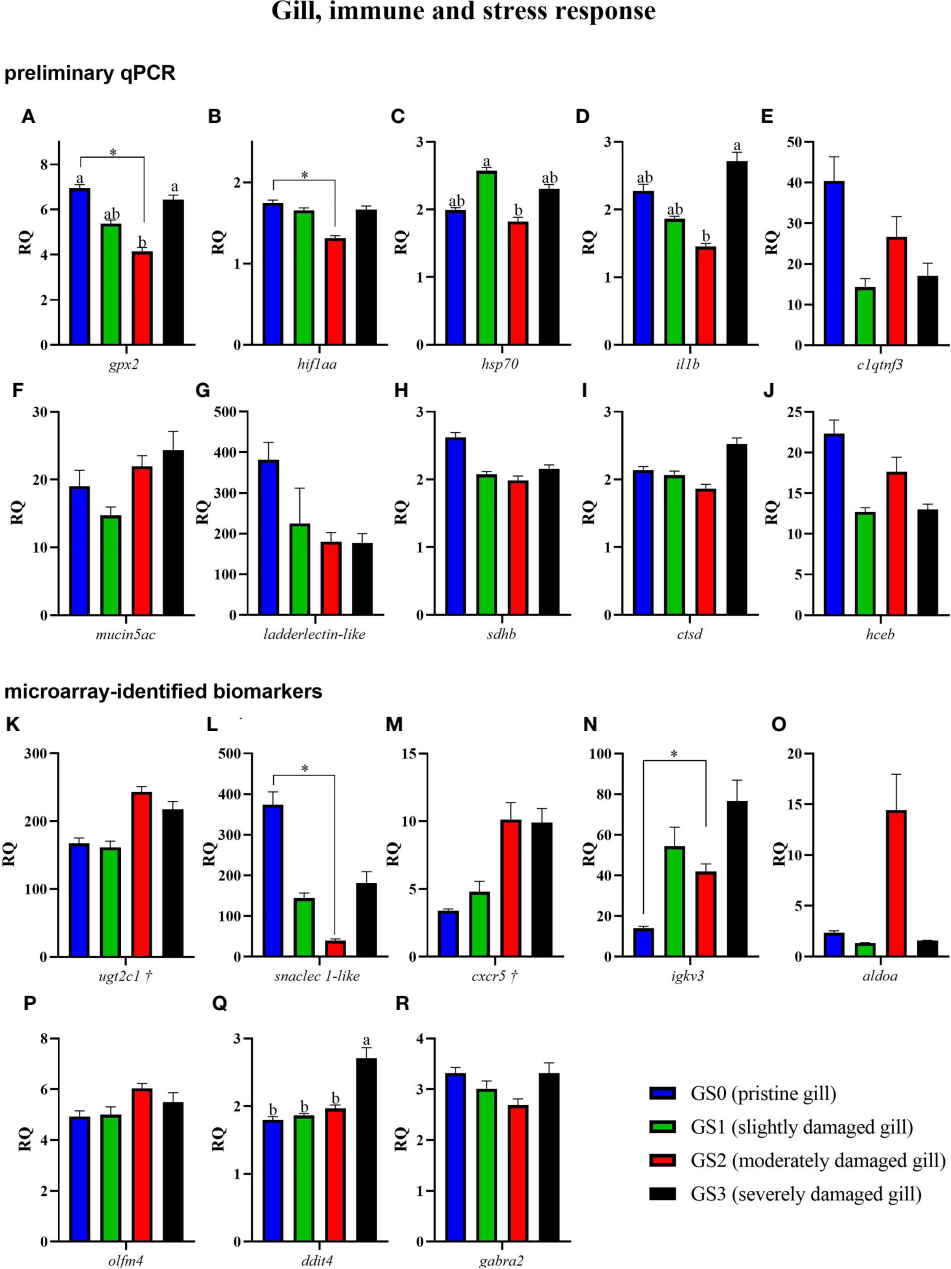
Bar plot of gill transcripts related to the immune and stress-relevant theme **(A–R)**. Different letters indicate a significant difference (p< 0.05) between groups using one-way ANOVA. Asterisks (*) are used to identify significance between GS0 and GS2 using t-test. Gene symbols followed by a dagger (†) are associated with p-values between 0.05 and 0.10 using t-test (GS0 vs GS2; i.e., *ugtc1* and *cxcr5*).

The preliminary (named as preliminary qPCR in **Figures 7**, **8**) and the confirmation qPCR (named as microarray-identified biomarkers in **Figures 7**, **8**) results are shown in **Figures 7**, **8**. Twelve GOI (as described in Section *Sample Selection Using qPCR*) were targeted in the preliminary qPCR and 27 GOI (i.e., microarray identified) were chosen to qPCR-validate the microarray results. The targeted transcripts for the qPCR confirmation of microarray-identified biomarkers were chosen based on their FC (i.e., highest FC values), representation of different pathways, and enriched GO terms. All of the collected gill samples, including GS0, GS1, GS2, and GS3, were included in the preliminary and qPCR confirmation studies.

In **Figure 7**, RQs of transcripts related to gill remodeling and wound healing were plotted (for both the preliminary qPCR and microarray-identified biomarkers). The preliminary qPCR results showed no significant difference between gill scores for *mmp19* and *mmp13b* (**Figures 7A, B**). The qPCR analysis of the microarray-identified biomarkers showed that the transcript levels of *des*, *neb*, *klh14b* and *abi3bp* (**Figures 7G, H, K, R**) were higher in GS2 than all other gill scores. The mRNA levels of *eln* and *myom1* were significantly higher in GS2 than GS1 and GS3 (**Figures 7I, U**). The levels of *pgam2* were higher in GS2 than GS1 (**Figure 7T**). Although there was high intragroup variability in the qPCR results, the microarray log_2_FC (identified by the RP analysis) showed significant positive correlation to qPCR log_2_ FC (calculated using each individual GS2 RQ value divided by the average GS0 RQ value; R=0.59, p-value<0.001; **Figure 7V**).

The RQs of transcripts related to immune and stress-relevant pathways were plotted in **Figure 8** (for both the preliminary qPCR and microarray-identified biomarkers). The preliminary qPCR results showed that *gpx2* mRNA expression was significantly higher in GS0 and GS3 compared with GS2 (**Figure 8A**). The levels of *hif1aa* were significantly lower in GS2 when compared with GS0 (t-test; **Figure 8B**), while the levels of *hsp70* were higher in GS1 when compared with GS2 (**Figure 8C**). The levels of *il1b* were lower in GS2 when compared with GS3 (**Figure 8D**). The qPCR results of the microarray-identified novel biomarkers showed that the transcript levels of *snaclec 1-like* mRNA were lower in GS2 compared with GS0 (t-test; **Figure 8L**). The transcript levels of *igkv3* were significantly higher in GS2 when compared with GS0 (t-test; **Figure 8N**). The levels of *ddit4* were significantly higher in GS3 compared with the three other gill scores (**Figure 8Q**). All the remaining genes did not have differential expression between groups.

### 3.5 Multivariate Analysis for All Gill RQs with p-Value ≤ 0.1 and Correlation Analyses

**Figure 9:**
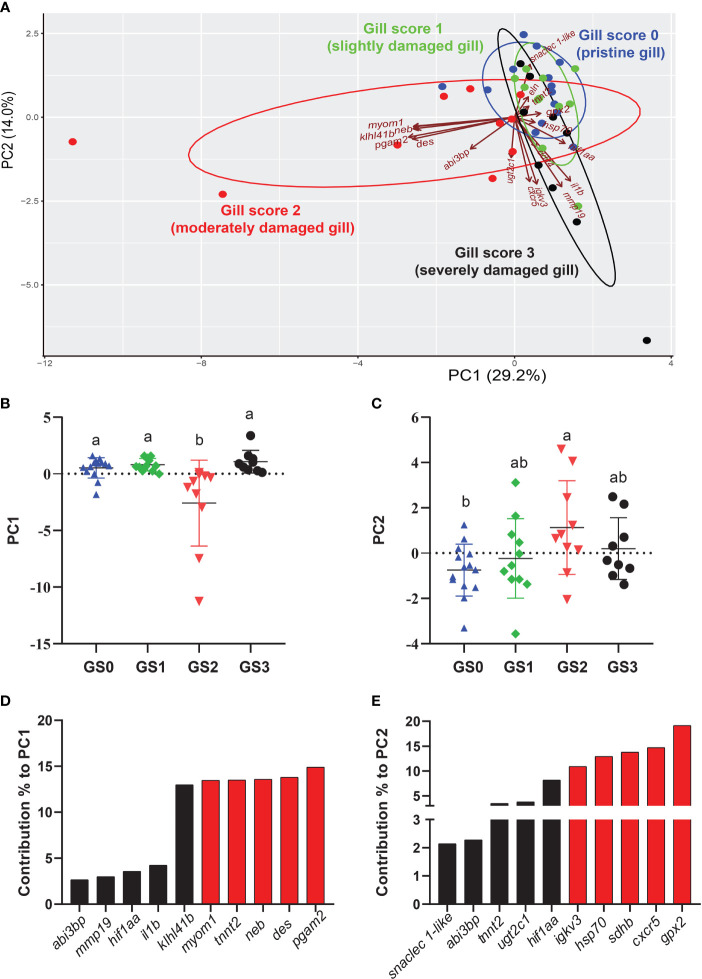
Principal component analysis (PCA) using gill RQ values of targeted transcripts with p-value≤ 0.1 between at least two gill scores. **(A)** PCA of individuals and vectors. The length and direction of arrows (vectors) indicate the loading of each transcript on the PC axis. **(B, C)** Scatter dot plots of PC dimensions 1 and 2 scores for individual samples. **(D, E)** Bar plot of the top ten variables contributing to each PC **(D, E)**. The top five contributing genes on each PC are highlighted in red.

The PCA was performed using RQs from all the investigated transcripts (preliminary and microarray-identified) with p-value < 0.1 studied in the gill tissue (**Figure 9**). PC1 explained 29.2% of the variance across groups, while PC2 explained 14.0% of the variance. The PC1 scores significantly separated GS2 from all other gill scores (p-value= 0.0003; **Figure 9B**). The top 10 transcripts contributing to this significant separation were plotted in **Figure 9D**. The highest five transcripts contributing to this separation were *pgam2*, *des*, *neb*, *tnnt2*, and *myom1* (**Figure 9D**). Individuals from GS0 and GS1 showed more overlap than GS0 and GS3. The PC2 significantly separated GS2 from GS0 (p-value=0.03; **Figure 9C**). The top ten transcripts contributing to this significant separation were plotted with the highest five contributors (i.e., *gpx2*, *cxcr5*, *sdhb*, *hsp70* and *igkv3*) highlighted in red (**Figure 9E**). A vector representing *snaclec 1-like* was plotted closer to GS0 and GS1 individuals. Vectors representing *cxcr5*, *igkv3* and *mmp19* were plotted negatively on PC2 (**Figure 9A**).

**Figure 10:**
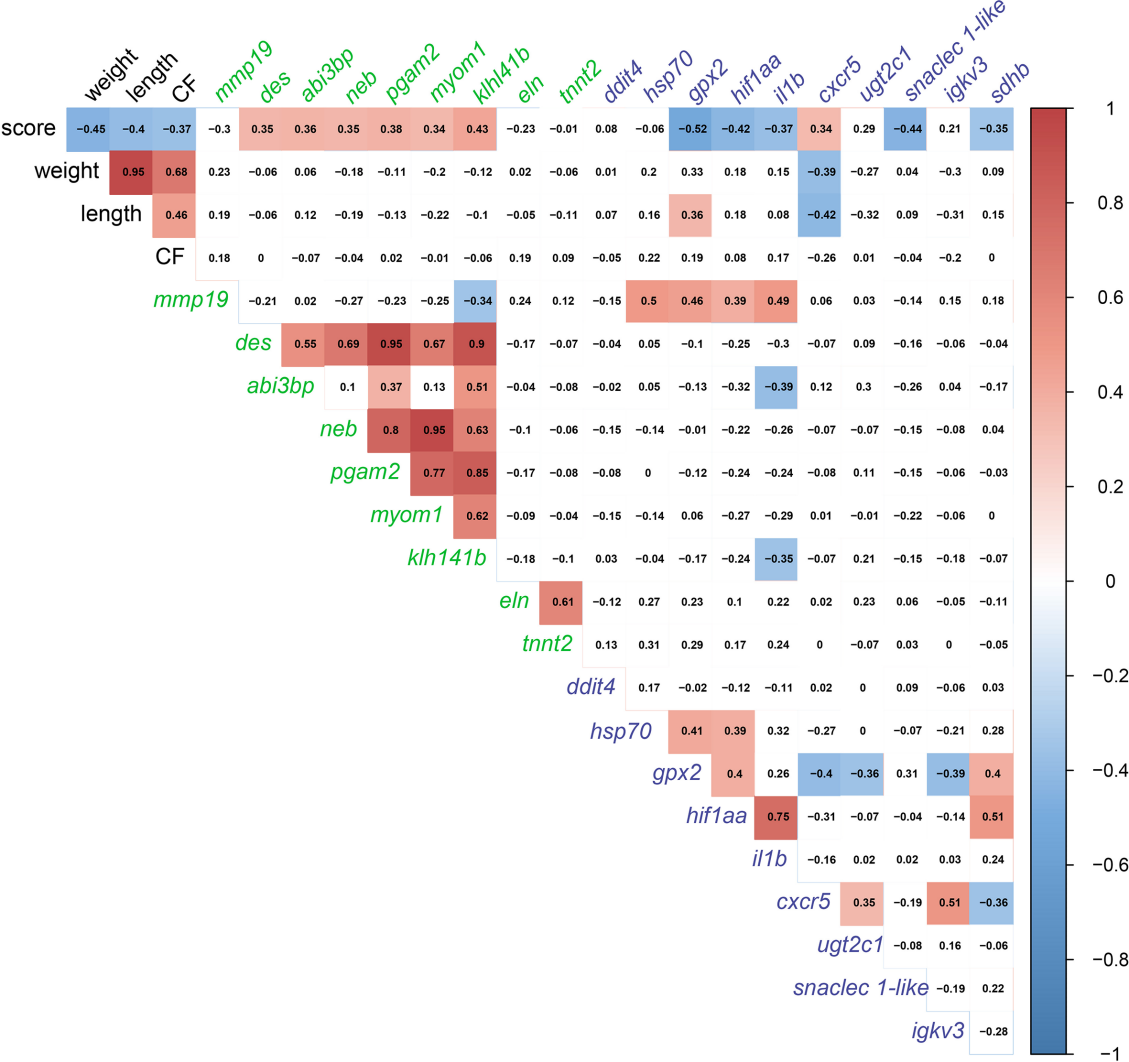
Correlation matrix using gill damage scoring (GS0, GS1, and GS2 only; GS3 individuals excluded), weight, length, condition factor (CF), and gill RQ values of targeted transcripts with p-value≤ 0.1 between at least two gill scores. Transcript names are colored based on the pathway (i.e., gill remodeling and wound healing in green; and immune and stress-relevant pathway colored in purple.

In order to detect significant correlations between transcript levels and the degree of gill damage (i.e., score), we excluded GS3 individuals from the correlation matrix (**Figure 10**). We attempted to include GS3 individuals in the correlation analysis, and no significant correlations were observed (data not shown). Also, the microarray-identified novel biomarkers were part of the moderately damaged (i.e., GS2) transcriptomic signature (in other words, GS3 individuals did not share in identifying those biomarkers). Gill scores (i.e., GS0-GS2) were significantly negatively correlated with weight, length and CF. Also, gill scores were positively correlated with gill remodeling and wound healing biomarkers (i.e., *des*, *abi3bp*, *neb*, *pgam2*, *myom1* and *klh141b*). Furthermore, gill scores were negatively correlated with most immune and stress-relevant biomarkers (i.e., *gpx2*, *hif1aa*, *il1b*, *snaclec 1-like* and *sdhb*), except for *cxcr5*, which showed a significant positive correlation with gill damage scores. Many wound healing biomarkers (i.e., *neb*, *pgam2*, *myom1* and *klh141b*) were positively correlated with one another. The levels of *eln* were correlated positively with *tnnt2*. In addition, stress-relevant biomarkers (i.e., *hsp70*, *gpx2* and *hif1aa*) were positively correlated with one another. *Cxcr5* was negatively correlated with *gpx2* and *sdhb*, and positively correlated with *ugt2c1* and *igkv3*.

### 3.6 Liver qPCR

**Figure 11:**
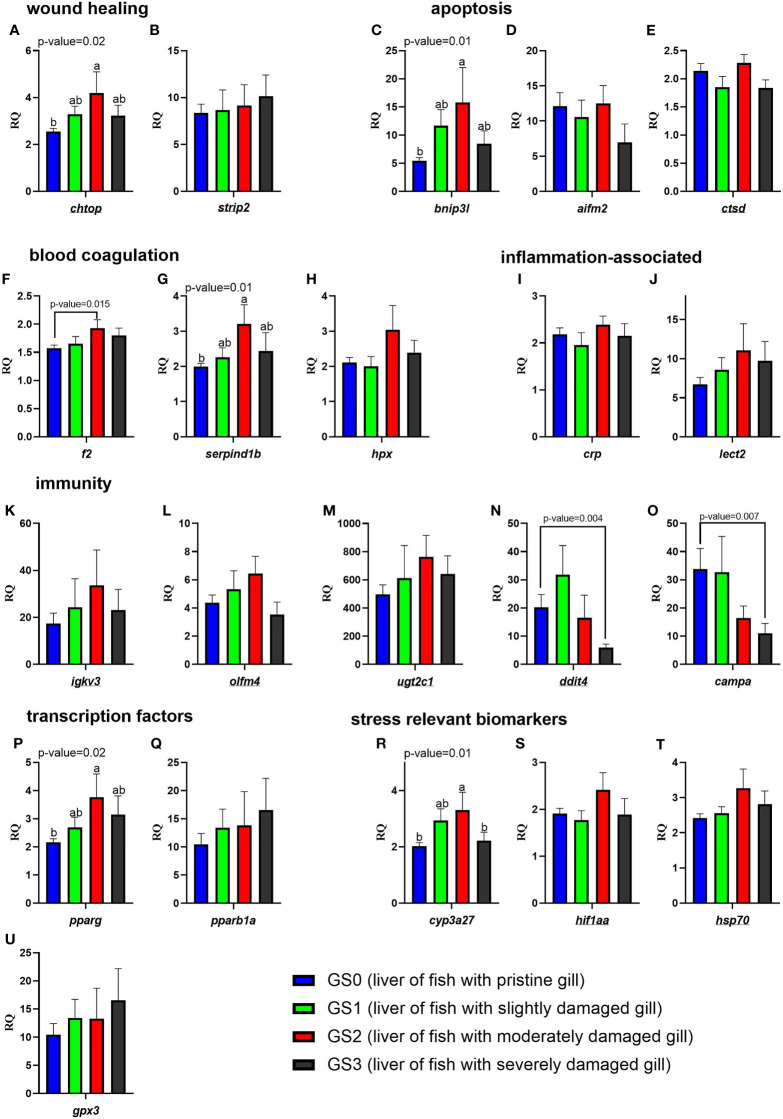
Bar plots of transcript expression levels of genes related to wound healing, apoptosis, blood coagulation, inflammation-associated proteins, immunity, transcription factors, and stress relevant biomarkers in the liver tissues of salmon with all four gill scores. The underlined gene symbols are the overlapping biomarkers between the gill and the liver qPCR. Different letters indicate a significant difference (p< 0.05) between groups using one-way ANOVA. Asterisks (*) are used to identify significance (p< 0.05) between GS0 and GS2, or between GS0 and GS3, using a t-test. Gene symbols are shown in the bottom of each figure panel **(A–U)**.

The RQs of the targeted transcripts in the liver are presented in **Figure 11**. In the functional categories of wound healing, apoptosis, and blood coagulation/APR, three genes (*chtop*, *bnip3l*, and *serpind1b*) were significantly upregulated (as shown by one-way ANOVA) in the liver of GS2 salmon compared with GS0 salmon with intermediate expression in GS1 and GS3 liver samples (**Figures 11A, C, G**). Using the t-test, the mRNA levels of *f2* were significantly higher in GS2 compared with GS0 (**Figure 11F**). In the functional category related to immunity, the transcript levels of *ddit4* and *campa* were lower (using T-test) in GS3 compared to GS0 (**Figures 11N, O**). In the transcription factor and stress-relevant categories, the mRNA levels of *pparg* and *cyp3a27* were higher (as shown by one-way ANOVA; **Figures 11P**, **11R**) in GS2 than GS0. Also, *cyp3a27* levels were higher in GS2 than in GS3 (**Figure 11R**). All the remaining genes did not have differential expression between groups.

### 3.7 Multivariate Analysis and Correlations for Liver

**Figure 12:**
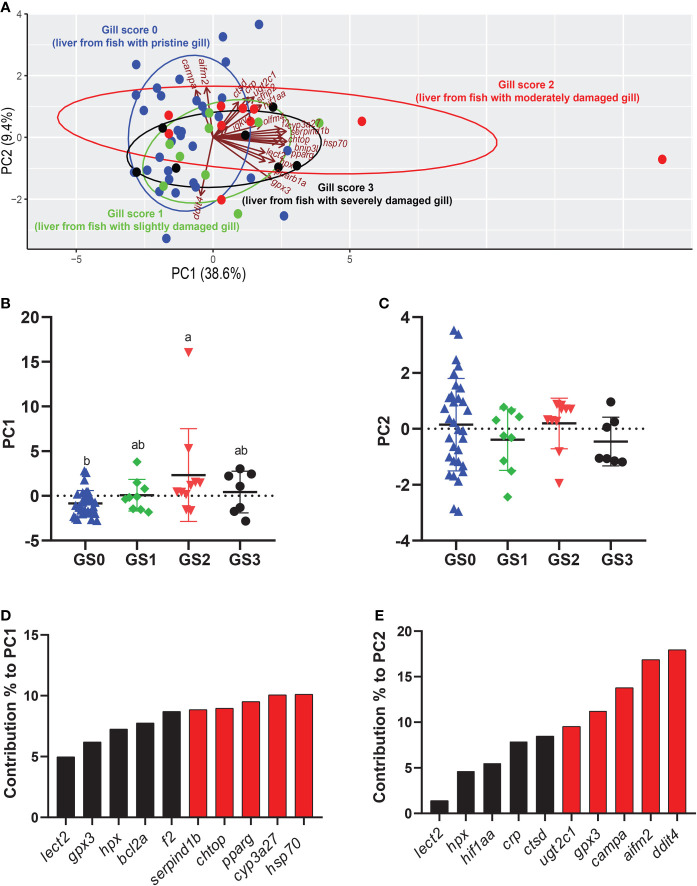
Principal component analysis (PCA) using all targeted transcript relative quantities (RQs) in the liver tissue. **(A)** shows a PCA of individuals and vectors. The length and direction of arrows (vectors) indicate the loading of each transcript on the PC axes. **(B, C)** Scatter dot plots of PC dimensions 1 and 2 scores for individual samples. **(D, E)** Bar plot of the top ten variables contributing to each PC. The top five contributing genes on each PC are highlighted in red.

The PCA was performed using all the targeted transcripts in the liver tissue (**Figure 12**). The PC1 explained 38.6% of the variance, while the PC2 explained 9.4% of the variance. The PC1 scores significantly separated GS2 from GS0 (p-value= 0.007; **Figure 12B**). The top 10 transcripts contributing to this significant separation were plotted, with the highest five transcripts contributed to this separation being *hsp70*, *cyp3a27*, *pparg*, *chtop* and *serpind1b* (**Figure 12D**). The PC2 did not significantly separate the gill score groups in terms of RQs of the targeted transcripts (**Figure 12C**). Vectors representing *cyp3a27*, *hsp70*, *bnip3l*, *pparg*, and *hpx* were plotted positively on PC1 (**Figure 12A**). The majority of GS2 individuals were plotted positively on PC1, while the majority of GS0 and GS1 individuals were plotted negatively on PC1 (**Figure 12A**).

**Figure 13:**
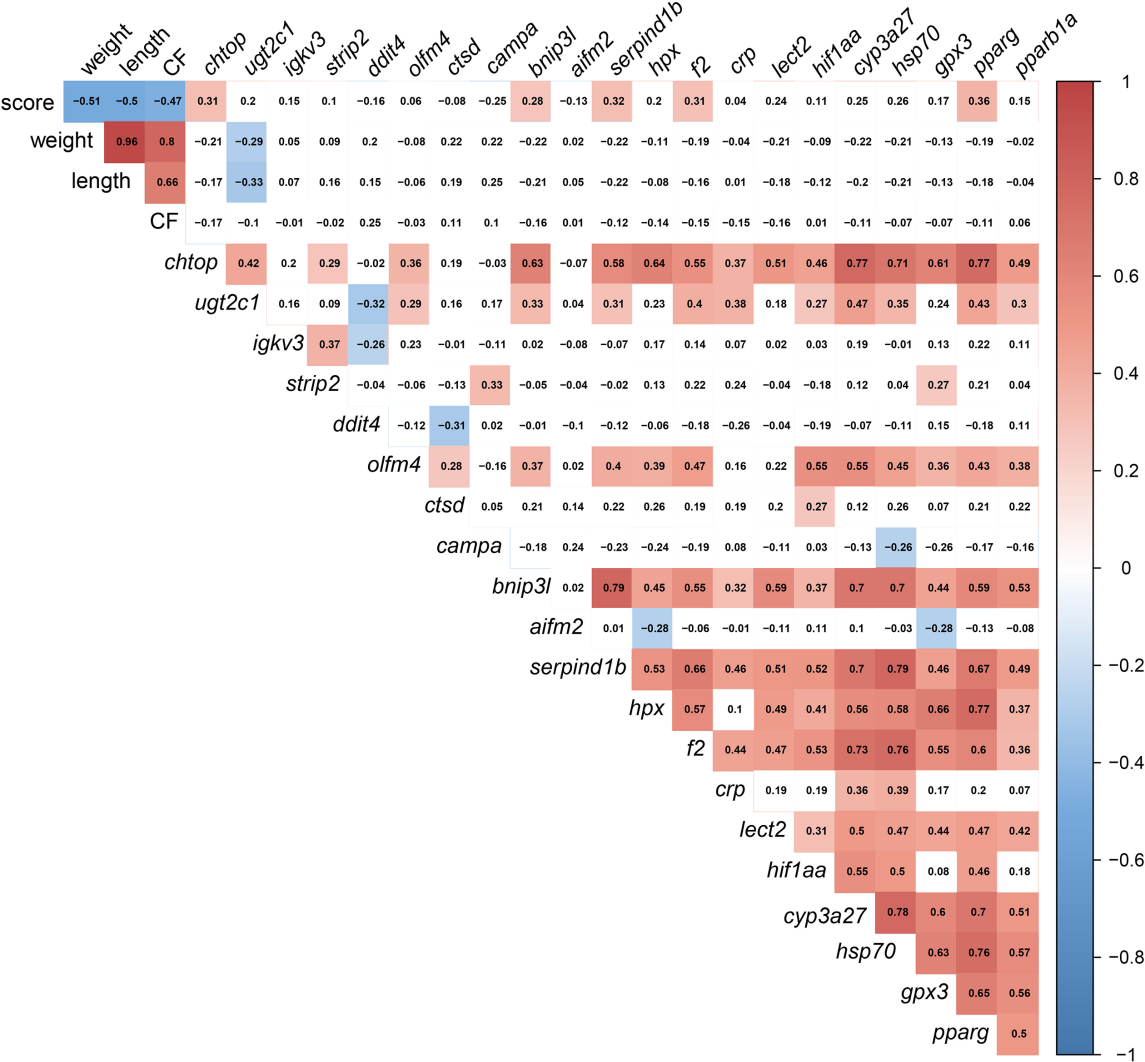
Correlation matrix using gill damage scoring, weight, length, condition factor (CF), and liver transcript expression data (RQ values). The significant correlations (p <0.05) were shaded in red (positive) or blue (negative), using all gill scores (i.e., GS0-GS3).

The significant correlations were shaded in red (positive) or blue (negative) in **Figure 13**, using all gill scores. To summarize, the gill score was positively correlated with *chtop*, *bnip3l*, *serpind1b*, *f2*, and *pparg* (**Figure 13**). Both *chtop* and *ugt2c1* were significantly and positively correlated with *olfm4*, *bnip3l*, *serpind1b*, *f2*, *crp*, *hif1aa*, *cyp3a27*, *hsp70*, *pparg* and *pparb1a.* In addition, the transcript levels of *chtop* were significantly positively correlated with *bnip3l*, *serpind1b*, *lect2, pparg*, and *cyp3a27*. The levels of *ugt2c1* and *igkv3* were negatively correlated with *ddit4*. Both *bnip3l* and *serpind1b* were positively correlated with *hpx, f2, crp, lect2, hif1aa, cyp3a27, hsp70, gpx3, pparg* and *pparb1a*. In addition, the transcript levels of *bnip3l* were positively correlated with *serpind1b*. Also, *hpx* was positively correlated with *f2*, *lect2*, *hif1aa*, *cyp3a27*, *hsp70*, *gpx3*, *pparg*, and *pparb1a*. The transcript levels of *cyp3a27*, *hsp70*, *gpx3*, *pparg*, and *pparb1a* were significantly and positively correlated.

## 4 Discussion

Compromised gill health [i.e., with regard to multifactorial stressors affecting gill tissue integrity (physical integrity assessed by visual inspection)] presents a significant challenge in the teleost fish aquaculture industry as it can lead to production losses and compromised fish welfare (10, 52, 53). This is supported in the current study, in which we demonstrated a negative correlation between level of gill damage and fish growth parameters (i.e., **Figure 13**). Of the causes of gill damage, HABs (e.g., blooms of *H. akashiwo*) are considered some of the main non-infectious threats to gill health (54). The transcriptomic response of damaged Atlantic salmon gill tissues impacted by multiple environmental stressors (e.g., HABs, elevated temperature) has not been well investigated. Moreover, Atlantic salmon in open-ocean aquaculture pens are exposed to highly variable and complex environments (15, 55). Research in gill transcriptome correlates of environmentally associated tissue damage may help identify relevant pathways and biomarker genes associated with multifactorial gill pathologies. The present study found many wound healing and immune/stress-related genes differentially expressed in moderately damaged gills (i.e., GS2; compared to healthy appearing gill, i.e., GS0) of Atlantic salmon grown in open-ocean pens.

Our qPCR analyses confirmed the microarray results as both datasets showed significant positive correlation using linear regression analysis (p-value<0.001; qPCR log_2_FC *vs*. RP log_2_FC; **Figure 7V**). However, for some of the microarray-identified genes [e.g., *calsequestrin 2* (*casq2*)], the qPCR analyses did not find a statistically significant response to moderate gill damage (i.e., GS2 *vs*. GS0; t-test). This lack of significance was likely caused by the addition of more biological replicates for GS2 and GS0 groups in the qPCR analysis (i.e., 14 GS0 and 10 GS2 in the qPCR study *vs*. 5 GS0 and 5 GS2 in the microarray study). Furthermore, the RP method is less sensitive to high biological variability than other statistical methods to detect differentially expressed probes within a microarray dataset (e.g., SAM) (30, 36, 38, 56). Interestingly, some of the genes found to be non-significantly altered in the qPCR analysis showed significant correlation with gill damage scores [e.g., *C-X-C chemokine receptor type 5* (*cxcr5*)] or contributed notably to gill group segregation in the PCA [e.g., *troponin T, cardiac muscle isoform-like* (*tnnt2*)].

### 4.1 Gill Remodeling and Wound Healing

More than 70% of the enriched GO terms were classified into the gill remodeling and wound healing theme (**Supplementary Table 2**), representing genes with functional annotations relevant to wound contraction, glycolysis, and extracellular matrix remodeling. In the next sections, the results related to these gill damage associated biological processes are discussed.

#### 4.1.1 Wound Contraction and Remodeling

A high proportion of enriched GO terms identified as responsive to moderate gill damage were related to tissue remodeling and wound healing (**Figure 5**). The GO terms “actomyosin” and “muscle filament sliding” were two of the most significantly enriched GO terms. Actomyosin is the actin-myosin complex forming the filaments responsible for muscle contraction. The contraction of the supracellular actomyosin ring around the wound is crucial for its closure, which allows the tissue to regain its structure and function (57–59). The “actomyosin” GO term was represented by gill damage-induced transcripts such as *actin*, *alpha skeletal muscle 2-like* (*acta2*), *actin, alpha cardiac-like* (*actc1*), *myosin heavy chain, fast skeletal muscle-like* (*myh*), and *tropomyosin beta chain* (*tpm2*). Although these transcripts were not statistically significant in the qPCR confirmation experiment (**Figures 7C–F**), they agreed in the direction of change (i.e., upregulation in GS2 compared with GS0, **Table 2**) and showed similar expression profiles (i.e., highest in GS2, lowest in GS1 and GS3, and intermediate in GS0). Similarly, another *myosin* (i.e., *myosin heavy chain 7*) was previously found to be upregulated in Atlantic salmon gills with moderate histopathology (8). Other actomyosin-related transcripts (e.g., *actin alpha skeletal muscle, tropomyosin alpha 3 chain-3*) were upregulated in Atlantic salmon skin at 22 and 33 days post sea lice (*Lepeophtheirus salmonis*) infestation (dpi) (60). In the same study, *myh* was lice-induced at 22 dpi but downregulated at 33 dpi. Differences between the current study and what was observed in (60), might be attributed to salmon’s skin gene expression response to *L. salmonis*, as it is was previously described as changing drastically during the parasite’s development (61); therefore, discrepancies with the present study can be expected. Overall, these previous investigations and the current study agree that actomyosin-associated genes are induced in Atlantic salmon’s mucosal tissues (i.e., gill and skin) in response to damage.

In addition to *actin*, *myos*in, and *tropomyosin*, other genes encoding proteins associated with the “muscle filament sliding” GO term were microarray-identified as differentially expressed in the current study. The mRNA levels of *desmin-like* (*des*), *nebulin* (*neb*), and *kelch-like protein 41b* (*klhl41b*) were significantly higher in GS2 than all the other gill scores (**Figure 7**), and constituted some of the top contributing biomarkers in separating GS2 fish from the rest on PC1 (**Figure 9D**). DES is a key protein of the intermediate filaments (IF) [a component of the cytoskeleton (62)] in skeletal, cardiac, and smooth muscle cells (63). Genes encoding IF proteins have been reported in mammals to be upregulated during the remodeling phase of wound healing (reviewed in (62)). DES binds to the sarcomere through its high affinity to NEB (64). NEB stabilizes muscle thin filaments structure (65), which is necessary for normal contraction. As well, it has been proposed that Kelch proteins like KLHL41 have a role in cytoskeletal organization, cell motility, and modulating cellular architecture (66, 67). The knockdown of *klhl41* in zebrafish resulted in skeletal muscle myopathy and reduced motor function (reviewed in (68)). Furthermore, the inhibition of KLHL41 in mice caused a fatal muscle sarcomere disarray and failure in NEB stabilization (69), which underlines its physiological importance. Here, *des*, *neb*, and *klhl41b* were positively correlated with one another and gill scores (**Figure 10**, which includes GS0, GS1, and GS2), suggesting that they might be co-regulated in response to moderate gill damage in Atlantic salmon.

The GO term “sarcoplasmic reticulum membrane” was a leading term within the wound healing and gill remodeling theme (**Figure 5**). In the current study, probes representing *casq2* and *klhl41b* contributed to this GO term enrichment. Despite the lack of statistical significance (p-value= 0.13; **Figure 7J**), qPCR results for *casq2* agreed with the microarray results in direction (GS2 fold-change > GS0 fold-change; **Table 2**). As mentioned above, *klhl41b* was significantly upregulated in GS2 compared with the other groups (**Figure 7K**). The sarcoplasmic reticulum (SR) is a membrane-bound structure within the muscle cell, with the primary function of storing calcium (70). CASQ2 acts as a buffer for calcium storage in the SR (70). Recently, fish sarcoplasmic proteins have attracted research interest as they are considered relevant proteins to wound healing (71). Further research is needed to understand the putative role of *casq2* in Atlantic salmon healing processes and evaluate its potential as Atlantic salmon gill healing biomarker gene.

*Myomesin 1* (*myom1*), contributed to the enrichment of many GO terms (i.e., 46 GO terms, e.g., “sarcomere” GO term; **Supplementary Table 2**). The qPCR results showed that the levels of *myom1* were significantly higher in GS2 compared with GS1 and GS3 (one-way ANOVA; **Figure 7U**). A trend of upregulation was observed comparing GS2 with GS0 (t-test; p-value= 0.08). Also, the PCA showed that *myom1* was one of the top contributing biomarkers to separate GS2 from all other gill scores individuals on PC1 (**Figure 9D**). MYOM is one of the required proteins incorporated in the assembly of the sarcomere, suggesting that the activation of this gene is involved in rebuilding and repairing the sarcomere after damage (72). Also, *myom1* expression levels have previously been proposed to detect muscle damage in zebrafish (*Danio rerio*) (72). Altogether, several transcripts encoding muscle structure and function-related proteins were differentially expressed, supporting the hypothesis that wound contraction is part of the proposed mechanism of healing in moderately damaged gill.

*Myh*, *tropomyosin beta chain* (*tpm2*), and *tnnt2* contributed to the enrichment of the GO term “regulation of ATPase activity” (**Supplementary Table 2**). The transcript levels of *tnnt2* showed a trend of upregulation in GS2 compared with GS0 individuals (p=0.07). Also, *tnnt2* was one of the top contributing biomarkers to separate GS2 individuals from all other gill scores on PC1 (**Figure 9D**). Atlantic halibut (*Hippoglossus hippoglossus*) *tnnt2* transcription regulation was suggested to be involved in calcium-driven muscle contraction during metamorphosis in this flatfish (73). Several *troponins* (e.g., *troponin T, cardiac muscle* and *troponin T, fast skeletal muscle*) were found downregulated with sea lice infestation in the skin of Atlantic salmon (74). Also, in human skin subjected to 10% lactic acid stinging test, Tropomyosin 1 (alpha) was found downregulated (75). The differences between the current study and the results reported in the skin of lice-infected Atlantic salmon (74), and in human skin (75), might be due to the nature of the examined tissues as well as the specific causes of the damage. Interestingly, *TNNT2* mRNA levels were suggested as a possible marker for wound age estimation in rats (76). However, whether the observed muscle-associated transcript expression is related to wound age would require further research (e.g., tank-based controlled trial) with gill tissues samples for analyses (e.g., histopathology; gene expression) at various time points.

#### 4.1.2 Metabolism During Wound Healing

Wound closure (e.g., through actomyosin contraction) was concurrent with elevated glycolysis during zebrafish larval wound healing and tail regeneration (77). In the current study, glucose-metabolism related genes, *phosphoglycerate mutase 2* (*pgam2*) and *aldolase a, fructose-bisphosphate 1* (*aldoa*), were microarray-detected as responsive to moderate gill damage. Only *pgam2* was qPCR confirmed; the transcript levels of *pgam2* were significantly higher in GS2 when compared with GS1 (one-way ANOVA, **Figure 7T**), and a trend of upregulation was observed when comparing GS2 to GS0 (t-test, p-value= 0.067). Also, the PCA showed that *pgam2* was the top contributing biomarker on separating GS2 individuals from all other gill scores on PC1 (**Figure 9D**). In pufferfish (*Takifugu fasciatus*) and in hybrid yellow catfish “Huangyou-1”, higher mRNA levels of *pgam2* and other glycolysis-related biomarkers were observed responding to hypoxia in the liver and brain tissues (78, 79), posing a question to whether the gill *pgam2* upregulation found in the present study points to impaired tissue oxygenation due to injury. Conversely, *pgam2* was downregulated in the skin of Atlantic salmon infected with sea lice (80). Again, the disagreement between the current study and the skin response to sea lice observed in (80) might be attributed to the host response to sea lice infestation, type of tissue (i.e., skin *vs*. gill) and/or age of wound. A member of the same family, *pgam5*, was found to encode a protein related to oxeiptosis, an anti-inflammatory-regulated cell death response to reactive oxygen species (ROS) (81). It should be noted that ROS, phycotoxins, and fatty acids might play an important role in ichthyotoxicity of *Chattonella marina*, a marine raphidophyte associated with red tides (82). To summarize, the present findings may suggest changes in glucose metabolism in the gill tissues as a result of damage.

#### 4.1.3 Extracellular Matrix Remodeling

In the current study, *elastin* (*eln*) was microarray-detected (**Supplementary Table 1**) and showed higher mRNA levels in GS2 compared with GS1 and GS3 according to the qPCR results (**Figure 7I**). Also, *eln* was associated with several enriched GO terms related to muscle development (e.g., muscle tissue development, striated muscle tissue development; **Supplementary Table 2**). Both collagen and elastin fibers (e.g., ELN) are important components in the extracellular matrix (ECM) and essential for skin integrity in mammals (83). ELN mitigates wound contraction and enables dermal regeneration (84). *Eln* was found upregulated in the damaged skin of Atlantic salmon infested with sea lice 33 dpi (60); alongside the current results, this collectively suggests that *eln* may respond to tissue damage irrespective of its cause.

A transcript related to cell movement and ECM binding, *abi family, member 3 [nesh] binding protein* (*abi3bp*), was microarray-identified and qPCR-validated as upregulated in GS2 compared with all other gill scores (**Figure 7R**). ABI3BP is an ArgBP/E3B1/Avi2/NESH family protein involved in inhibiting cell movement and metastasis (85, 86). It promotes cellular senescence and cell-ECM binding interactions (87). ABI3BP downregulation by miR-183 suppressed proliferation, activity, and migration of human esophageal cancer cells (88). Research is required to investigate ABI3BP's potential function during tissue healing processes in fish.

Although *matrix metalloproteinases* (*mmps*) were not identified by the microarray (GS2 *vs*. GS0), we included *matrix metalloproteinase-19* (*mmp19*) and *matrix metalloproteinase-13 b* (*mmp13b*) during the preliminary qPCR (**Figures 7A, B**) because they were previously found differentially expressed with damage (8, 29). The levels of *mmp19* showed a trend (t-test; p-value=0.07) of downregulation in GS2 *vs*. GS0, whereas *mmp13b* showed no significant response or trend. MMPs play a key role in skin wound healing; however, prolonged dysregulation (although we do not have evidence that this is the case in the current study) of *mmps* might lead to hindered wound healing and persistent inflammation (89). In Atlantic salmon, while sea lice infection caused *mmp19* downregulation in the fin (29), moderate gill histopathology showed induced expression of *mmp13* (8). The mammalian literature regards MMP19 as an ECM-degrading enzyme involved in wound-healing (90); however, MMP19 might be involved in immune-related function (91). In mice, MMP19 showed involvement in epithelial cell migration (92) and cutaneous T-cell development (91). Furthermore, in the current study *mmp19* was positively correlated with four stress and immune relevant genes (i.e., *hsp70*, *gpx2*, *hif1aa*, and *il1b*) and negatively correlated with one potential remodeling related gene (i.e., *klhl41b*) (**Figure 10**). This might indicate its involvement in both gill damage repair and immune and stress responses.

### 4.2 Immune and Stress Response Theme

The second theme identified in the current study is the immune and stress response. In this theme, the leading GO terms were “tertiary granule lumen”, “detection of chemical stimulus”, “cell-cell recognition”, “phagocytic vesicle membrane”, and “immunoglobulin complex, circulating” (**Figure 5**). It should be noted that wound healing (as a general term) is a complex biological process that does not separate between remodeling (or tissue restoration and wound closure) and the inflammatory/immune response. For example, tertiary granules are typically found in activated human neutrophils and contain cathepsins and metalloproteinases, which mediate their migration and their pro-inflammatory and antimicrobial activity (93, 94).

More than 25% of the enriched GO terms were classified in the immune and stress response theme (**Supplementary Table 2**), representing genes with functional annotations relevant to innate and adaptive immune responses, as well as stress responses. In the next sections, the results related to these gill damage associated biological processes are discussed.

#### 4.2.1 Stress-Relevant

In the current study, several probes representing members from the heat shock protein family (known as stress proteins and extrinsic chaperones, reviewed in (95)) were found to be upregulated (i.e., *hspb1* and *dnajb6* (alias *hsp40*)) in GS2 compared to GS0 (**Supplementary Table 1**). During the preliminary qPCR, we also targeted stress-relevant biomarkers *glutathione peroxidase 2-like* (*gpx2*) and *hypoxia-inducible factor 1 alpha* (*hif1aa*), and they were significantly downregulated in GS2 compared with GS0 (t-test; **Figures 8A, B**). Also, both *gpx2* and *hif1aa* were significantly negatively correlated with gill scores (**Figure 10**, not including GS3). The transcript levels of another stress-relevant biomarker, *heat shock protein* 70 (*hsp70*), were significantly lower in GS2 than in GS1 (**Figure 8C**; preliminary qPCR) and were significantly positively correlated with those of *gpx2* and *hif1aa* (**Figure 10**). Also, *gpx2* and *hsp70* were top contributors in significantly separating GS2 from GS0 on PC2 (**Figure 9E**). In mammals, vascular damage can create a hypoxic microenvironment at the injured tissue site, prompting the induction of HIF-1 (96). However, the observed *hif1a* downregulation in the current study seems not to support the tissue hypoxia hypothesis but rather may suggest HIF1A signaling pathway involvement in promoting tissue fibrosis (96, 97). Furthermore, the local stabilization of *HIF-1* promoted mammalian intestinal epithelial healing and controlled intestinal inflammation (98). Therefore, this might be part of a molecular mechanism to prevent excessive scarring, promoting healing and preserving gill function (97, 99).

The glutathione peroxidase family has a well-known antioxidant function through the reduction of hydrogen peroxide, which is involved in different signaling mechanisms (e.g., apoptosis, cell differentiation, and proliferation) (100). GPX2 knockout mice showed increased apoptosis at colonic crypt bases (101). Apoptosis promotes cell removal in wounded or infected tissue (102). Therefore, *gpx2* downregulation in the moderately damaged salmon gills may have been part of a molecular mechanism to promote apoptosis. Previously, Król et al. (8) reported *glutathione peroxidase 6-like* upregulation in Atlantic salmon gills with moderate histopathology. Altogether, the current study microarray results (i.e., upregulation of *hspb1* and *dnajb6*), and the preliminary qPCR results (i.e., dysregulation of *gpx2*, *hif1aa* and *hsp70*), together with Król et al. (8), might suggest that there may be different responses of stress-relevant biomarker genes between aquaculture sites/environments, i.e., dependent on multiple factors such as different stressors causing or predisposing damage, wound chronicity, and salmon epigenetics and population. However, all the mentioned results did not explore the progression of the healing process (i.e., represent snapshots of an overall process).

The GO term “detection of chemical stimulus” was enriched in our list of moderate gill damage-responsive genes (**Figure 5**), among which we found *UDP-glucuronosyltransferase 2C1* (*ugt2c1*). The transcript levels of *ugt2c1* showed a trend of upregulation in GS2 compared with GS0 (p-value=0.076). In mammals, glucuronidation reactions catalyzed by UGT are important to detoxify lipophilic compounds in the liver (103). The recorded HAB data prior to the gill sampling showed the salmon in the present study were exposed to high cell concentrations of ichtyotoxic microalgae like *Heterosigma akashiwo* and *Chrysochromulina* sp (104, 105) (**Figure 2D**). Even during non-toxic HAB events, lysed algal cells can release metabolites (e.g., free fatty acids and free radicals), causing gill damage in open-ocean aquaculture fish (106). Also, needle-shaped diatoms can physically damage and clog fish gills (17), which might facilitate toxin entry. The significant upregulation in the microarray and the qPCR trend of *ugt2c1* upregulation in GS2 *vs*. GS0 led us to consider that algal toxins could be one of the contributing factors to the observed gill damage.

Microarray and qPCR analyses found *snaclec 1-like* (snake C-type lectins-like, named based on the proposed nomenclature in (107)) to be significantly downregulated in GS2 compared with GS0 (**Supplementary Table 1**, **Figure 8L**). Also, it was found to be negatively correlated with gill score (**Figure 10**). *Snaclec*, together with other genes encoding toxins, were identified during the genome assembly of the Chinese yellow catfish (*Pelteobagrus fulvidraco*) (108). Genes encoding toxins (e.g., *Snaclec*) in animals undergo more accelerated evolution than non-toxin related genes (109). However, the functional characterization of those genes in teleosts has not been well studied (108). SNACLECs produced by snakes can affect hemostasis and thrombosis, and may alter the normal function of endothelial and smooth muscle cells, keratinocytes, and inflammatory processes by promoting the overproduction of pro-inflammatory cytokines (110, 111). Considering the bioactivity of such proteins in snakes, the molecular function of fish produced SNACLECs and involvement in gill damage warrants future research.

In the current study, *DNA damage-inducible transcript 4* (*ddit4*) was significantly induced only in severely damaged gills (i.e., GS3 *vs*. the rest) (**Figure 8Q**). Mammalian *Ddit4* has been identified as a responsive gene to UV-induced DNA damage (112, 113), as well as oxidative stress, hypoxia, and endoplasmic reticulum stress (114–116). Furthermore, it has been suggested that DDIT4 might induce autophagy and apoptosis through the mTOR signaling pathway in cardiomyocytes (117). In red seabream, *Pagrus major*, DNA damage and oxidative stress were reported due to *Cochlodinium polykrikoides* dinoflagellate during a HAB (118). Also, *ddit4* was found upregulated in the muscle of red cusk-eel (*Genypterus chilensis*) in response to thermal stress (119). Taken together, the upregulation of *ddit4* in GS3 gills aligns well with our environmental observation suggesting the salmon were exposed to a series of HAB episodes (**Figure 2**; **Supplementary Table 3**). Also, its involvement in apoptosis and/or autophagy suggests those pathways may be part of the gill response to severe damage, which further justifies our concern of including GS3 individuals in the microarray study.

#### 4.2.2 Immune Response

The transcript levels of *interleukin 1 beta* (*il1b*) were significantly upregulated in GS3 when compared with GS2. Also, *il1b* showed a trend towards downregulation in GS2 compared with GS0 (t-test; p-value= 0.087). IL1B is a well-known cytokine secreted by activated phagocytes (e.g., macrophages) to trigger an inflammatory response in the surrounding tissue in response to injury or infection (120–122). Also, *il1b* was found upregulated, in the fins of Atlantic salmon infested with sea lice (29). The observed difference in direction of *il1b* transcript expression response between GS2 *vs*. GS0 and GS3 *vs*. GS0 might indicate the gill’s inflammatory response is regulated based on the degree of the damage.

According to our qPCR results, the mRNA levels of the microarray-identified *cxcr5* did not differ significantly among gill score groups. Nevertheless, *cxcr5* expression levels were positively correlated with gill damage (**Figure 10**), as it showed a trend of upregulation in GS2 compared with GS0 (p-value= 0.068). Moreover, *cxcr5* was one of the top contributors that significantly separated GS2 from GS0 on PC2 (**Figure 9E**). Chemokines play a crucial role in various stages of the healing and immune processes (123, 124). Chemokine signaling pathways are proposed as a therapeutic target to decrease wound fibrosis, chronic wound development, and pathological scarring (125). Also, CXCR5 and its ligand CXCL13 act on the recruitment of B and T lymphocytes trafficking to and within secondary lymphoid tissue (126). *Cxcr5* was previously characterized and found expressed in the gill tissue of grass carp (*Ctenopharyngodon idella*) (127). The observed correlation of gill damage (i.e., GS0- GS2) might suggest the involvement of CXCR5 in the protection against pathogens in slightly-to-moderately damaged gill.

The microarray experiment found 39 enriched GO terms related to immunoglobulin-mediated processes (**Supplementary Table 2**) and 126 probes representing immunoglobulins were upregulated in GS2 compared with GS0 (**Supplementary Table 1**). For qPCR confirmation, a transcript representing *ig kappa chain V-III region mopc 63*, *igkv3* [best hit of GenBank accession number was 96.61% identical to “BT046734.1”] was targeted. The transcript levels of *igkv3* were higher in GS2 compared with GS0 (**Figure 8N**; t-test). Also, *igkv3* was one of the top contributors separating GS2 from GS0 on PC2 (**Figure 9E**). Immunoglobulins (IGs) are essential for adaptive mucosal immunity as reviewed in (128). Microbe detection in the fish gill mucosa induces B cells’ immunoglobulin production in GIALTs (129–131). Also, IG (i.e., IgT as the predominant IG induced in the gill mucosa) response in the gill tissue to parasitic (i.e., *Ichthyophthirius multifiliis*) and bacterial (i.e., *Flavobacterium columnare*) infection were previously reported in rainbow trout (*Oncorhynchus mykiss*) (132). Immunoglobulins are known to neutralize pathogens and promote their elimination in the mucosa (133). Damaged gills (e.g., due to HAB) can act as a port of entry for pathogens, which might induce IGs production to neutralize the pathogens before they establish infection. Additionally, the GIALT role in producing algal toxin-neutralizing antibodies has not been well studied (134).

Two microarray probes representing *cd209 antigen-like protein c* (*cd209*c) and *matrix-remodeling-associated protein 5-like* (*mxra5*) were upregulated in GS2 compared with GS0. CD209 is a Ca^2+^-independent C-type lectin-like receptor that recognizes a wide range of pathogens (e.g., viruses, bacteria, and parasites) and participates in activating T and B lymphocytes (135). CD209 has been reported as a marker for anti-inflammatory M2 macrophages, in large yellow croaker (136). MXRA5 has anti-inflammatory and anti-fibrotic properties in mammals (137). The observed consistent upregulation of anti-inflammatory biomarkers in moderately damaged gills may suggest the activation of mechanisms aiming to mitigate inflammation.

### 4.3 Liver qPCR

In order to study the systemic response associated with different gill damage scores, we targeted the liver. The liver was an appropriate organ for this study as it is considered as a key systemic regulator for relevant processes [e.g., APR (including blood coagulation, inflammation-associated), apoptosis, and immune response; **Figure 11**]. In salmonids, liver physiology has been found to respond to infections by skin ectoparasites (60), pathogenic bacteria (138) and viruses (139), as well as exposure to toxicants (e.g., hydrogen peroxide (140), chromium (141)), high temperatures, and hypoxia (142). Therefore, we aimed to investigate the liver response associated with gill damage by qPCR-analyzing the expression levels of a selection of biomarker genes putatively involved in wound healing, apoptosis, APR, immunity, and stress response. The selection of these genes of interest was guided by the gill microarray DEP and well-known biomarkers related to the aforementioned biological processes.

#### 4.3.1 Acute Phase Response (Including Blood Coagulation and Inflammation-Associated) and Apoptosis

Several biomarkers with putative roles in the APR (143–145) were targeted in the liver, classified to “blood coagulation”, and “inflammation-associated” in **Figure 11**. Some of those biomarkers showed significant positive correlation to damage (i.e., score; **Figure 13**; including all gill scores GS0-GS3) and to each other [i.e., *heparin cofactor 2 B* (*serpind1b*) and *prothrombin* (*f2*; alias: *coagulation factor II*)]. Also, *hemopexin-like* (*hpx)* was significantly positively correlated with *f2* and *leukocyte cell derived chemotaxin 2* (*lect2*). This indicates the relevance of the hepatic APR to gill damage.

Genes encoding proteins involved in the coagulation cascade were found differentially expressed in the liver tissue of salmon of different gill scores. Two genes related to blood clotting, *serpind1b* and *f2*, presented higher mRNA levels in GS2 than GS0 (**Figures 11F, G**). Also, the PCA showed that *serpind1b* was one of the top contributors separating GS2 from GS0 in PC1 (**Figure 12D**). While coagulation was initially considered to be a transient and acute response (as part of the APR (144, 146)) during tissue injury (143), the coagulation cascade is also involved in subsequent wound healing stages (e.g., inflammatory and fibro-proliferative responses) (147). Members of the serpin family are known for their ability to inhibit serine proteases involved in regulating different biological processes such as hemostasis and inflammatory responses (148). Also, plasma SERPIND1 is considered to be part of the APR signaling pathway (144). Prothrombin can be cleaved to form the activated serine protease thrombin (149), which catalyzes fibrinogen conversion into fibrin once it reaches blood circulation, activates platelets, and increases endothelial permeability to stop blood loss at the site of injury (150). The consistent upregulation of these two coagulation-relevant biomarkers and their significant correlation with gill score suggest hepatic assistance in salmon gill repair. These results suggest potential applications of coagulant-enhancing therapeutics and feed additives (e.g., vitamin K) in the development of clinical diets designed to mitigate moderate gill damage.

The hepatic transcript levels of *bnip3l* (gene encoding the protein known as BCL2/adenovirus E1B 19 kDa protein-interacting protein 3) were upregulated in GS2 compared with GS0 (**Figure 11C**). BNIP3L is a member of the Bcl-2 family and one of the pro-apoptotic proteins that induce apoptosis, necrosis, or autophagy (151, 152). BCL2 family proteins are known to regulate both mitochondrial physiology and cell death in mammals (151). Apoptosis plays a major role in inducing a cascade of biochemical events that changes the cell morphology and leads to cell death (153). Fibroblast apoptosis was previously reported during wound healing (154). In mammals, autophagy is induced by BNIP3 through the Jun N-terminal kinases (JNK), mitogen-activated protein kinases (MAPK), and hypoxia-induced ROS-mediated p38 stimulation, which promotes the migration of epidermal keratinocytes during wound healing (155). Also, the APR was reported to induce apoptosis (156). This is supported with significant positive correlation of *bnip3l* with several APR biomarkers (e.g., *serpind1b*, *hpx*, *f2*, and *lect2*). The relevance of hepatic *bnip3l* upregulation to both apoptosis and/or autophagy pathway(s) with APR during gill damage in in teleosts requires further study. Nevertheless, mammalian and piscine *bnip3l* are known to be responsive to environmental stressors (e.g., hypoxia (157, 158)). Rainbow trout embryos showed increased mRNA levels of various *bnip3l* paralogues after being exposed to hypoxic stress for 24 h, an effect that seemed to persist throughout development [at least until fry stage (159)]. Interestingly, liver *bnip3l* was significantly positively correlated with several stress-relevant biomarkers (e.g., *hif1aa*, and *hsp70*), which might further draw attention to the possible systemic impacts associated with hypoxia as a resultant of gill damage. However, *hif1aa* (a well-known hypoxia biomarker) transcription did not respond to gill damage; further research is necessary to test these hypotheses.

#### 4.3.2 Hepatic Stress Response

The hepatic transcript levels of *cytochrome P450 3A27 B* (*cyp3a27*) were higher in GS2 than GS0 (**Figure 11R**). Also, the liver PCA showed that *cyp3a27* was one of the top contributors in separating GS2 individuals from GS0 on PC1 (**Figure 12D**). Cytochrome P450 (CYP) is a heme-containing enzyme superfamily that has a major role in metabolizing foreign compounds (e.g., pollutants and drugs) (160). Several *cytochrome p450s* (including *cyp3a27*) were found expressed in the liver tissue of rainbow trout (*O. mykiss*) (161). In rainbow trout, CYP3A27 was capable to metabolize steroid hormone (160). Furthermore, *cyp3a27*, together with other pregnane X receptor relevant genes, were upregulated in the liver of rainbow trout exposed to the insecticide chlorpyrifos (162). Moreover, CYP3A27 was increased in the liver of coho salmon (*Oncorhynchus kisutch*) with salinity acclimation (163). Changes in the expression of genes encoding enzymes related to metabolite clearance (e.g., *cyp3a27*) might modulate the liver’s ability to clear toxicants (164). In support of a hepatic toxin clearance activation hypothesis associated with gill damage (as a port of entry), monitoring the phytoplankton composition and abundance at the farm site prior to sampling revealed the blooms of *Heterosigma akashiwo* and *Chrysochromulina* sp., which are known for producing toxins (165). Several stress-relevant transcripts qPCR-analyzed in the present study (e.g., *cyp3a27*, *hif1aa*, and *hsp70*) were significantly positively correlated with one another, thus suggesting co-regulation and/or possible response to environmental changes. The question remains open for several hypotheses (e.g., whether these genes responded to toxins from phytoplanktonic algae; gill function impairment concurrent with possible tissue hypoxia).

#### 4.3.3 Hepatic Immune Response and Inflammation

The levels of *ddit4* and *cathelicidin A* (*campa*) were significantly lower in GS3 than GS0 (**Figures 11N, O**; t-test). As previously mentioned in section *Immune and Stress Response Theme*, DDIT4 may be involved in apoptosis induction (117) through its regulatory activity of mitochondrial function (166). Furthermore, DDIT4 acts as a negative regulator of mammalian rapamycin mTOR (114), which mediates in the mounting of the innate defense response (167). Cathelicidins are short cationic peptides that are known for their immunomodulatory functions (168). In teleost fish, cathelicidins may regulate transcripts with pro-inflammatory relevant function (e.g., some interleukins), however, this is species and cell type specific (169). Furthermore, they showed antibacterial and IL8 stimulating activity in salmonid species (170). Also, cathelicidins were reported as leukocyte chemoattractants in mice (171), and they might contribute to the inflammatory process through the activation of mast cells to release histamine (172). Cathelicidin-NV, a member of the cathelicidin family, showed a wound-healing promoting activity by directly enhancing keratinocyte proliferation to accelerate epithelization and fibroblast to myofibroblasts differentiation for wound contraction in mice (173). The observed downregulation of hepatic *ddit4* and *campa* in GS3 fish of the current study might suggest a possible immunomodulatory role of the liver tissue during the response to severe gill damage (i.e., GS3) (e.g., modulating inflammation at the damaged gill tissues). This is further supporting the previously proposed hypothesis with *pparg* upregulation. Taken together, the systemic (i.e., liver) response concomitant with gill damage warrants a high-throughput transcriptomic study to elucidate concurrent dysregulated pathways.

The transcription factor *peroxisome proliferator-activated receptor-gamma* (*pparg*) was found significantly upregulated in the liver of the moderately damaged (i.e., GS2) compared with GS0 (**Figure 11P**). Also, it was one of the main contributors in separating GS2 from GS0 on PC1 (**Figure 11D**), and it was positively correlated with gill damage scores (**Figure 13**). PPARG is a nuclear receptor that belongs to the nuclear hormone receptor family and is responsible for regulating the expression of genes involved in the homeostasis of glucose, lipid metabolism, and regulation of cell growth, inflammation, and connective tissue biology (159). Also, PPARG plays a key role in immune defense and anti-inflammatory mechanisms (174), as it is involved in the inhibition of NF-κB, AP1, and STAT transcription factors (175). In orange-spotted grouper (*Epinephelus coioides*), *pparg* was found upregulated with *Vibrio alginolyticus* challenge and the administration of PPARG antagonist (GW9662) upregulated the expression of pro-inflammatory cytokines (e.g., *il1b*, *il6*) (174). In Atlantic salmon a novel allele of *pparg* was associated with salmon resistance to *Aeromonas salmonicida* (176). The *pparg* upregulation in the current study might be part of a hepatic response to regulate systemic inflammation or maintain whole-body homeostasis in salmon with moderate gill damage. Due to known *pparg* functions, significant correlation with damage and several APR relevant genes (e.g., *serpind1b*, *f2, hpx*; **Figure 13**), and its hepatic induction in salmon with moderately damaged gills, this gene could be an important biomarker in future research aimed at developing gill healing-promoting therapeutics.

The liver qPCR results showed that the transcript levels of *chromatin target of PRMT1 protein-like* (*chtop*) were higher in GS2 than GS0 (**Figure 11A**). Also, the PCA showed that *chtop* was one of the top contributing biomarkers separating GS2 individuals from GS0 on PC1 (**Figure 12D**), and it was significantly correlated with gill damage score (i.e., including all gill scores; **Figure 13**). Interestingly, *chtop* was microarray identified as downregulated in the gill tissue, but it did not show significant difference in the gill qPCR confirmation (**Figure 7**). CHTOP is an intracellular protein that regulates the transcriptional activation of several oncogenic genes in mammals (177). CHTOP knockdown reduced the migration and the invasion of malignant ovarian cancer cell lines (177). The observed upregulation in GS2 in the current study might suggest the involvement of *chtop*’s encoded protein in the systemic response of the liver during gill damage, possibly influencing cellular migration as recorded in (177). Although cell recruitment at the wound site is an essential step during wound healing, there is a paucity of information on the liver’s involvement in cell recruitment at the wound site (e.g., gill) in teleosts.

## 5 Conclusion

The present study highlighted dysregulated pathways and biomarkers in moderately damaged gill tissues of Atlantic salmon farmed in open-ocean nets and exposed to environmental stress (possibly HABs, based on environmental data collected). Gill damage would likely impact its function, and consequently salmons’ welfare and growth performance. This study identified pathways that could be classified into two themes: 1) gill remodeling and wound healing, and 2) immune and stress response. The list of wound healing-related genes differentially expressed in the damaged gills was dominated with muscle structure and/or contraction relevant biomarkers, supporting the wound contraction hypothesis, and showed some overlap with previous findings in Atlantic salmon skin healing. Future comparative studies of wound healing across mucosal tissues could be valuable for fish aquaculture research and production. Immunoglobulin-mediated processes dominated the list of immune pathways responding to the gill damage. A limitation of the current study is that it did not screen for pathogens (e.g., infection or carrier state) at the time of tissue sampling for transcript expression analyses. We suggest that future transcript expression studies related to salmon gill health include pathogen screening, as the correlation of pathogen presence with gill damage and associated biomarker gene expression may be valuable in developing therapeutic strategies. Moderate gill damage also provoked the repression of well-known hypoxia (*hif1aa*) and oxidative stress (*gpx2*) biomarker genes, which may refer to the putative participation of their encoded proteins in healing processes (based on the mammalian literature). Finally, some gill damage-induced genes (e.g., *ugt2c1*) supported the hypothesis the observed gill damage may have been associated with previous toxic algal blooms.

The liver response of the explored biomarkers (e.g., APR relevant biomarkers, *pparg*) showed a more robust correlation with all gill scores (i.e., GS0, GS1, GS2, and GS3), compared with the gill transcriptional response correlation to the severity of gill damage (i.e., only included GS0, GS1, and GS2). As demonstrated by the present study and Król et al. (8), transcriptomics of diseased gills from fish in open-ocean aquaculture remains a challenging area of research due to multiple reasons (e.g., many potential stressors and pathogens causing gill damage; progression of wound development, resolution and severity). Notwithstanding, the study of the gill transcriptome changes throughout the healing process in fish warrants further investigation. The environmental stressors and predominant pathogens vary over time (e.g., seasonality) and space (i.e., from a fish aquaculture site to another) (178). Multi-site studies and meta-analyses will be necessary to identify common and site/experiment-specific gill gene expression responses to various combinations of stressors/infections. The present study represents one of the first steps towards a better understanding of gill damage (arising from complex environment) and provides resources (e.g., biomarker genes and associated qPCR assays) that will be valuable in the development of gill health-promoting strategies for the Atlantic salmon aquaculture industry.

## Data Availability Statement

The datasets generated for this study can be found in online repositories. The names of the repository/repositories and accession number(s) can be found at: https://www.ncbi.nlm.nih.gov/geo/, GSE186728.

## Ethics Statement

The animal study was reviewed and approved by internal and external committee following the Canadian Council on Animal Care.

## Author Contributions

Conceptualization: ME, MLR, AC-S, RB, and RT. Microarray analysis: ME, XX, and MLR. QPCR analyses: ME, XX, and MLR. Data interpretation: ME, XX, AC-S, NU, and MLR. Sample collection: AC-S, XX, and NU. Gill scoring: BM. Writing: ME. Manuscript reviewing and editing: ME, AC-S, MLR, RB, XX, and NU. Figure preparation: ME, AC-S, and MLR. Supervision: MLR. All authors contributed to the article and approved the final manuscript.

## Funding

ME is a post-doctoral fellow supported by the Mitacs Accelerate program (Grant 213950: co-sponsored by Cargill, Incorporated). The majority of research consumables were also funded by the Mitacs and Cargill, Incorporated co-funded grant through the Mitacs Accelerate program. The field study was part of a Genomic Applications Partnership Program projects [GAPP #6607: Integrated Pathogen Management of Co-infection in Atlantic salmon (IPMC) project] funded by the Government of Canada through Genome Canada and Genome Atlantic, as well as EWOS Innovation (now part of Cargill, Incorporated) to MLR. During the sampling both AC-S and NU were recipients of Mitacs Accelerate Postdoctoral Fellowships. The IPMC project was also funded by the Government of Newfoundland and Labrador through the Department of Tourism, Culture, Industry and Innovation (Leverage R&D award #5401-1019-108). Additional funding to MLR was provided by Natural Sciences and Engineering Research Council of Canada Discovery Grants (NSERC; 341304- 2012 and 2020-04519) and by the Ocean Frontier Institute, through an award from the Canada First Research Excellence Fund. Some of the primers for qPCR were designed during GAPP Biomarker Platform for Commercial Aquaculture Feed Development (project # 6604).

## Conflict of Interest

Authors RB, and RT, in the representation of Cargill, Incorporated, and BM, in representation of Cermaq, participated in the design of the trial but had no role in the design of the gene expression experiment, the data collection and analysis, the preparation of this manuscript, and the decision to submit the manuscript for publication. RT and BM are currently not working in Cargill, Incorporated and Cermaq, respectively. Also, NU is currently working at AquaBounty Canada, Inc.

The remaining authors declare that the research was conducted in the absence of any commercial or financial relationships that could be construed as a potential conflict of interest.

## Publisher’s Note

All claims expressed in this article are solely those of the authors and do not necessarily represent those of their affiliated organizations, or those of the publisher, the editors and the reviewers. Any product that may be evaluated in this article, or claim that may be made by its manufacturer, is not guaranteed or endorsed by the publisher.

## Supplementary Material

Supplementary Figure 1 Recorded environmental and mortality data, **(A) **Time series morning data of water temperature, salinity, and oxygen saturation. **(B)** Time series afternoon data of water temperature, salinity, and oxygen saturation data. **(C) ** Time series of the concentration (cells/mL) of various microalgae species potentially harmful for farmed Atlantic salmon, recorded at the farm site from June–November 2017. **(D)** Recorded fish mortalities. Mortalities were classified into different categories depending on the putative cause of death; i.e., mortalities attributed to environmental stress (e.g., algal blooms, hypoxia events) were classified as “Environmental”; mortalities suspected to be caused by *Tenacibaculum maritimum* infection (causative agent of mouth rot disease in salmonids; note: infection was not analytically confirmed) were annotated as “Mouth rot”; salmon euthanized due to their poor growth performance were designated as “Non-performers”; fish carcasses too deteriorated to be classified were named “Old”. Water temperature, salinity, oxygen saturation, and microalgae concentration were measured at 1, 5, 10, and 15 m depth. Water temperature, salinity, and oxygen saturation measurements were taken twice daily (6-9 am and 12-5 pm).

Supplementary Figure 2 Example of gill condition noted with environmental insults, named as “Environmental” mortalities in **Supplementary Figure 1**.

Supplementary Figure 3 Linear discriminant analysis (LDA) using preliminary qPCR relative quantities (RQs) on gill tissue for sample selection. Samples with bolded identifiers were selected for the microarray experiment.

Supplementary Figure 4 Schematic diagram showing the current study experimental design. This figure was constructed using BioRender (https://biorender.com/).
