# Supplementary figures and images for: Gill and Liver Transcript Expression Changes Associated With Gill Damage in Atlantic Salmon (Salmo salar)
Source: Front Immunol. 2022 Mar 28;13:806484. doi: 10.3389/fimmu.2022.806484 (PMC8996064; doi:10.3389/fimmu.2022.806484)

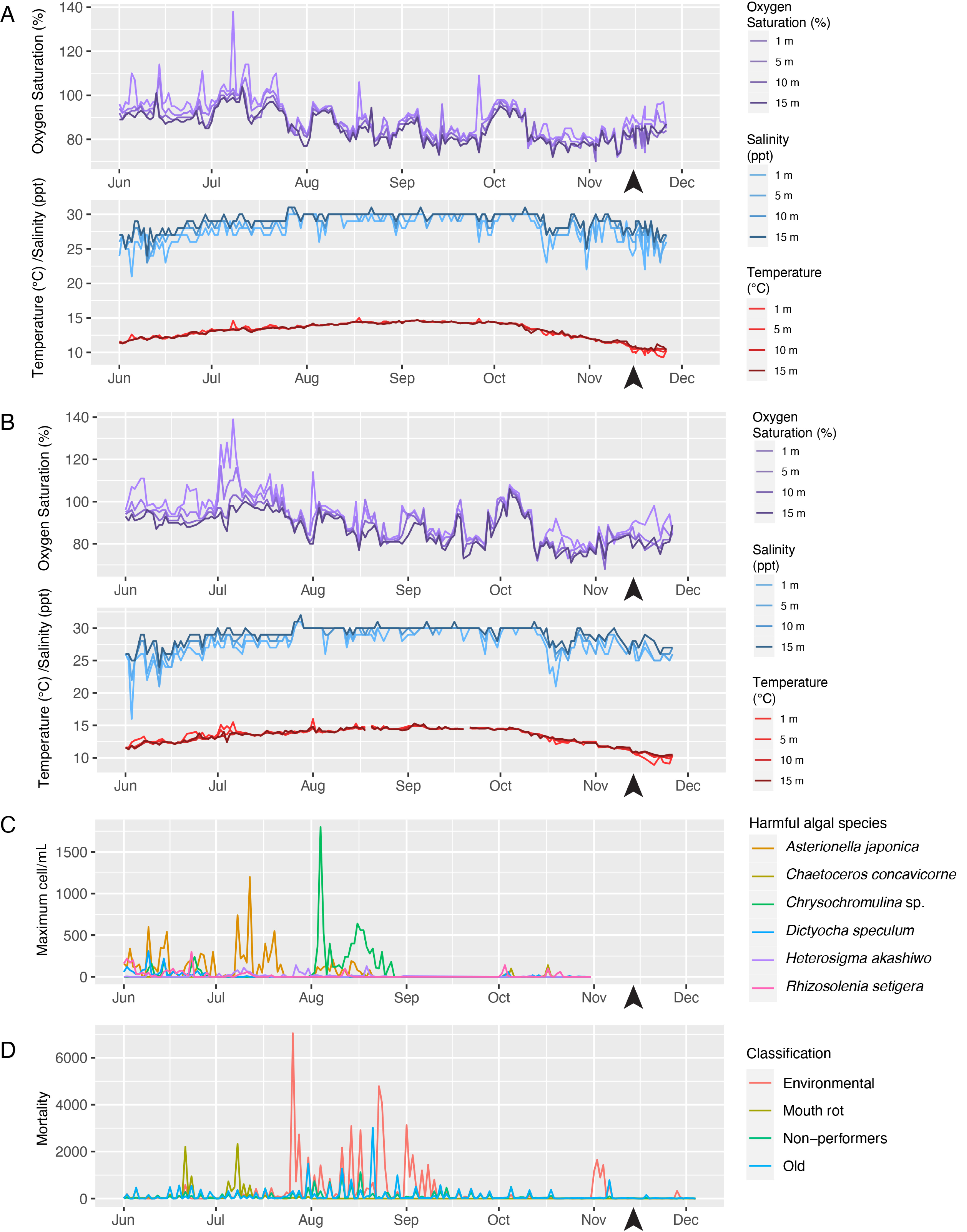

Supplement: Supplementary Figure 1 — Recorded environmental and mortality data, (A) Time series morning data of water temperature, salinity, and oxygen saturation. (B) Time series afternoon data of water temperature, salinity, and oxygen saturation data. (C) Time series of the concentration (cells/mL) of various microalgae species potentially harmful for farmed Atlantic salmon, recorded at the farm site from June–November 2017. (D) Recorded fish mortalities. Mortalities were classified into different categories depending on the putative cause of death; i.e., mortalities attributed to environmental stress (e.g., algal blooms, hypoxia events) were classified as “Environmental”; mortalities suspected to be caused by Tenacibaculum maritimum infection (causative agent of mouth rot disease in salmonids; note: infection was not analytically confirmed) were annotated as “Mouth rot”; salmon euthanized due to their poor growth performance were designated as “Non-performers”; fish carcasses too deteriorated to be classified were named “Old”. Water temperature, salinity, oxygen saturation, and microalgae concentration were measured at 1, 5, 10, and 15 m depth. Water temperature, salinity, and oxygen saturation measurements were taken twice daily (6-9 am and 12-5 pm). [file Image_1.jpeg]

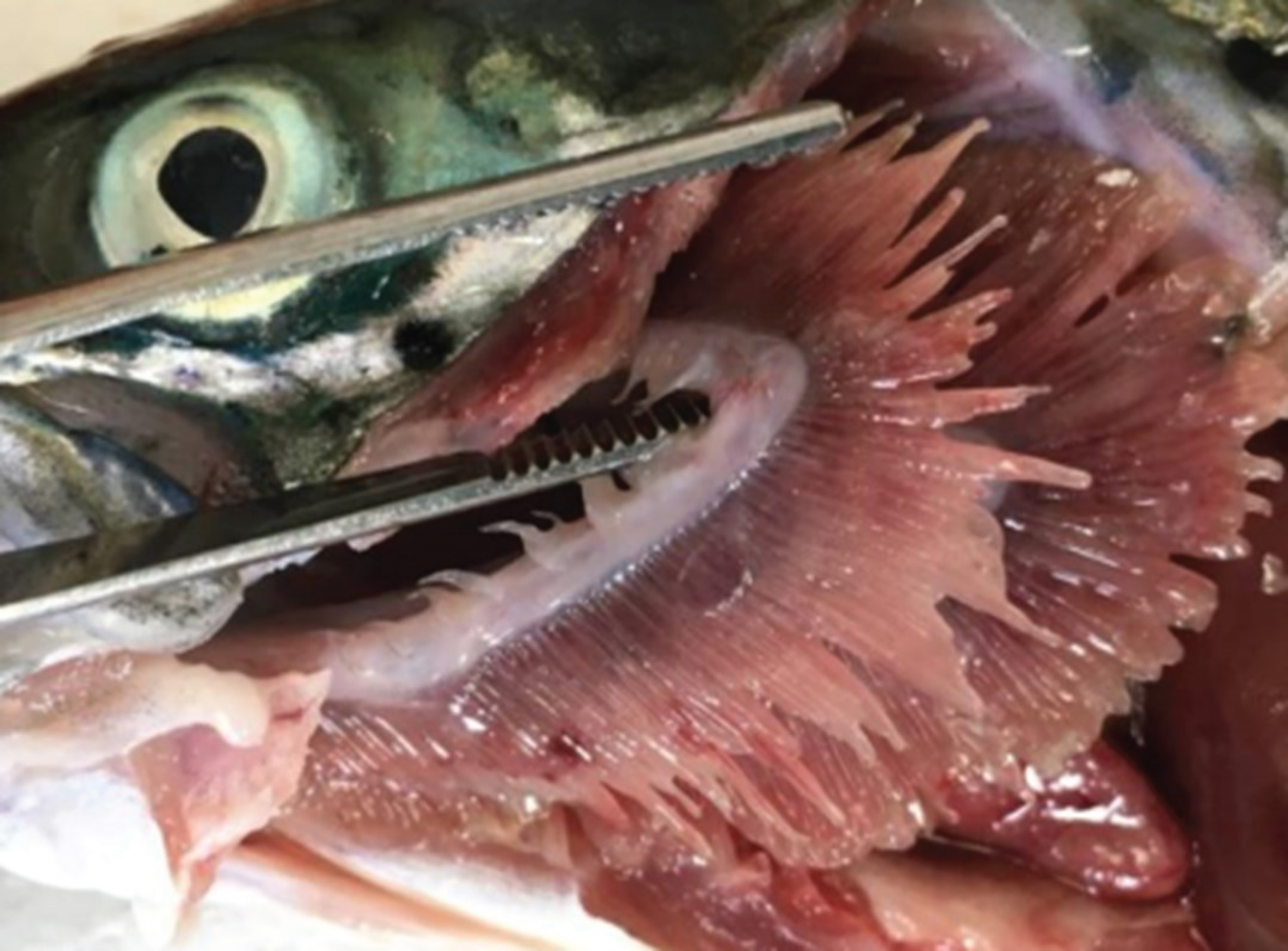

Supplement: Supplementary Figure 2 — Example of gill condition noted with environmental insults, named as “Environmental” mortalities in Supplementary Figure 1 . [file Image_2.jpeg]

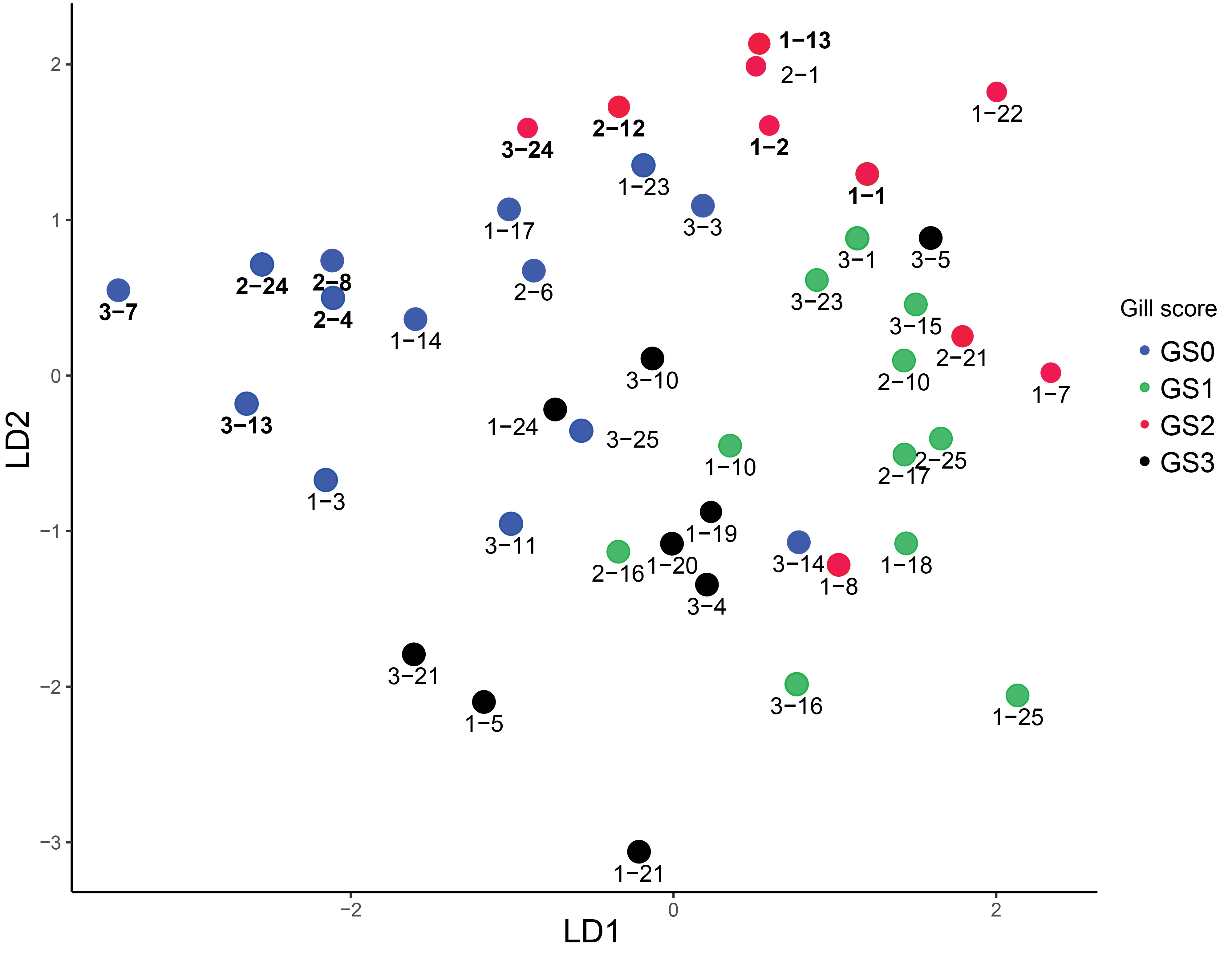

Supplement: Supplementary Figure 3 — Linear discriminant analysis (LDA) using preliminary qPCR relative quantities (RQs) on gill tissue for sample selection. Samples with bolded identifiers were selected for the microarray experiment. [file Image_3.jpeg]

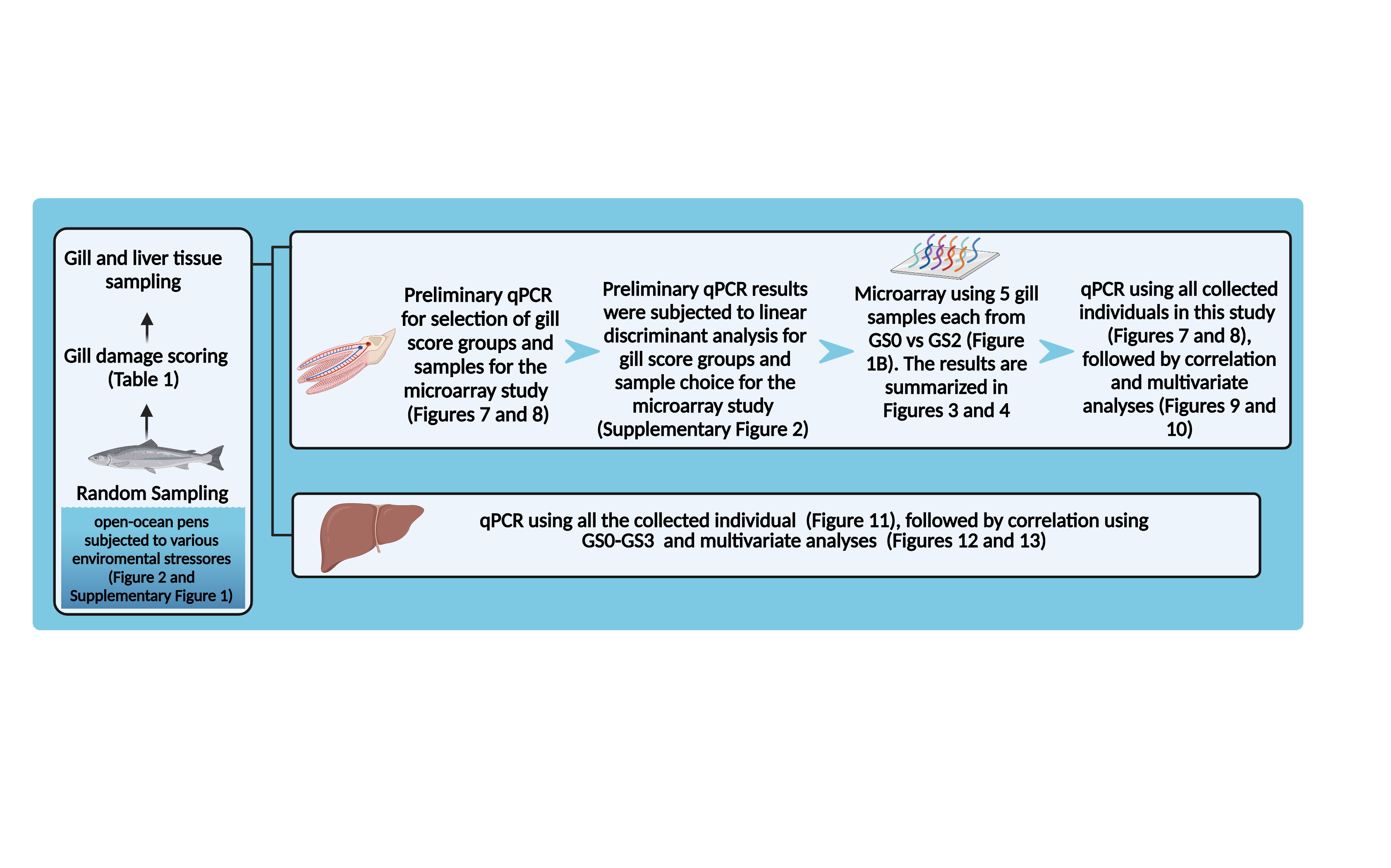

Supplement: Supplementary Figure 4 — Schematic diagram showing the current study experimental design. This figure was constructed using BioRender (https://biorender.com/). [file Image_4.jpeg]
